# Low Cystatin C–to–Creatinine Estimated GFR Ratio Predicts Post–Kidney Transplant Cardiovascular Events

**DOI:** 10.1016/j.ekir.2025.08.029

**Published:** 2025-08-26

**Authors:** Michael Moryoussef, Antoine Morel, Louai Zaidan, Soraya Fellahi, Cédric Usureau, Alain Luciani, Anissa Moktefi, Nizar Joher, Cécile Maud Champy, Philippe Grimbert, Marie Matignon, Florence Canouï-Poitrine, Frédéric Pigneur, Thomas Stehlé

**Affiliations:** 1 Institut National de la Santé et de la Recherche Médicale U955, Institut Mondor de Recherche Biomédicale, Universite Paris Est Créteil, Créteil, France; 2 Assistance Publique des Hôpitaux de Paris, Hôpitaux Universitaires Henri Mondor, Service de Néphrologie et Transplantation, Innovative Therapy for Immune Disorders Fédération Hospitalo-Universitaire, Créteil, France; 3 Assistance Publique des Hôpitaux de Paris, Hôpitaux Universitaires Henri Mondor, Service d’Imagerie Médicale, Créteil, France; 4 Institut National de la Santé et de la Recherche Médicale, Centre de Recherche Saint-Antoine UMR S_938, Institut Hospitalo-Universitaire de Cardio-Métabolisme et Nutrition, Sorbonne Université, Paris, France; 5 Assistance Publique des Hôpitaux de Paris, Hôpitaux Universitaires Henri Mondor, Département de Biochimie-Pharmacologie-Biologie Moléculaire-Génétique Médicale, Créteil, France; 6 Assistance Publique des Hôpitaux de Paris, Hôpital Saint Louis, Laboratoire d'Immunologie et Histocompatibilité, Paris, France; 7 Institut National de la Santé et de la Recherche Médicale UMR976, Institut de Recherche Saint-Louis, Université de Paris, Paris, France; 8 Assistance Publique des Hôpitaux de Paris, Hôpitaux Universitaires Henri Mondor, Service d’Anatomopathologie, Créteil, France; 9 Assistance Publique des Hôpitaux de Paris, Hôpitaux Universitaires Henri Mondor, Service d’Urologie, Créteil, France; 10 Assistance Publique des Hôpitaux de Paris, Hôpitaux Universitaires Henri Mondor, Service de Santé Publique & Unité de Recherche Clinique, Créteil, France

**Keywords:** cardiovascular events, creatinine, cystatin C, eGFR, myosteatosis, sarcopenia

## Abstract

**Introduction:**

A low cystatin C–based to creatinine-based estimated glomerular filtration rate (eGFR) ratio (eGFR_cys_/eGFR_cr_) is associated with poor clinical outcomes, but data in kidney transplant recipients (KTRs) remain limited. We aimed to investigate the association between low eGFR_cys_/eGFR_cr_ and allograft loss, major adverse cardiovascular events (MACEs) and mortality, and to identify the reasons for such a low ratio.

**Methods:**

This single-center retrospective study included KTRs with eGFR_cys_/eGFR_cr_ determined 3 months posttransplant using the Chronic Kidney Disease-Epidemiology Collaboration (CKD-EPI) and the European Kidney Function Consortium (EKFC) equations. Body composition data were obtained from a computed tomography (CT) slice at the third lumbar vertebra as follows: skeletal muscle mass index (SMI: cross-sectional muscle area divided by height squared), myosteatosis (mean muscle CT attenuation), as well as subcutaneous and visceral adipose tissue indexes.

**Results:**

Among 385 KTRs, an eGFR_cys_/eGFR_cr_ < 0.7 was found in 106 (27.5%) and 54 (14%) patients with the CKD-EPI and EKFC equations, respectively. A low eGFR_cys_/eGFR_cr_ ratio was independently associated with MACEs whatever the equation considered (adjusted hazard ratio [aHR]: 2.46; 95% confidence interval [CI]: 1.24–4.87 for CKD-EPI, and aHR: 2.29; 95% CI: 1.11–4.72 for EKFC), but not with death or allograft loss. Using CKD-EPI equations, diabetes mellitus was associated with eGFR_cys_/eGFR_cr_ < 0.7; using EKFC equations, diabetes mellitus and male sex were associated. In the 275 KTRs with available CT scan, male sex and myosteatosis were associated with low eGFR_cys_/eGFR_cr_ ratio, together with low SMI for CKD-EPI equations, and diabetes mellitus for EKFC.

**Conclusion:**

A low eGFRcys/eGFRcr in KTRs is associated with MACEs. Diabetes mellitus, male sex, myosteatosis, and low muscle mass could be key factors of such a low ratio.

## Graphical abstract

**Figure:**
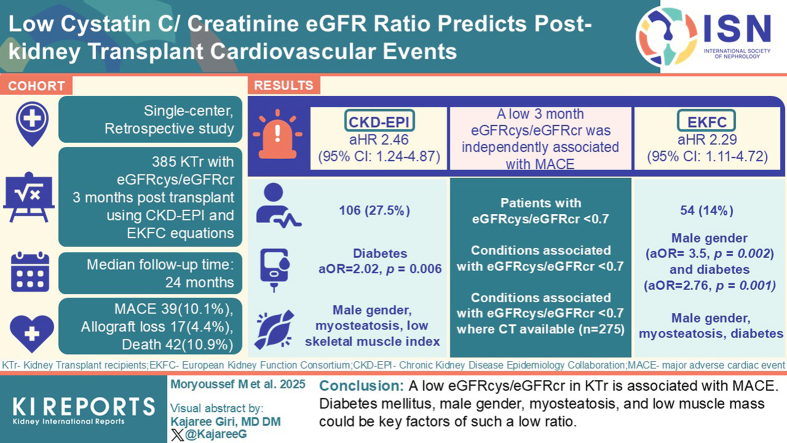

Glomerular filtration rate (GFR) is used to assess kidney function. In clinical practice, GFR is estimated (eGFR) with equations based on demographic data and endogenous serum or plasma biomarker levels, the most commonly used being creatinine. Cystatin C, a low molecular weight metalloprotease (13.3 kDa) synthetized by all nuclear cells and freely filtered by glomeruli, is used as an endogenous biomarker of GFR and has recently taken on greater importance. Indeed, the National Kidney Foundation and the American Society of Nephrology^1^ as well as the Kidney Disease Improving Global Outcomes guidelines^2^ have recently promoted the use of cystatin C in assessing eGFR, because equations combining the 2 biomarkers are more accurate when compared with measured GFR.^3^^,^^4^ This combination could provide a better categorization of patients according to their outcomes.^5^ Serum and plasma levels of creatinine and cystatin C are influenced by non-GFR determinants, for example, muscle mass, tubular secretion, protein intake for creatinine; or for example dysthyroidism or high-dose corticosteroid therapy for cystatin C.^6^ Several recent studies have shown that a low ratio between eGFR based on cystatin C (eGFR_cys_) and eGFR based on creatinine (eGFR_cr_) is associated with worse clinical outcomes such as frailty, cardiovascular events, as well as all-cause and cardiovascular mortality.^7^, ^8^, ^9^, ^10^, ^11^ In the field of kidney transplantation (KT), studies have focused on the non-GFR determinants of creatinine and cystatin C,^12^ the respective relationships of GFR_cys_ and GFR_cr_ with allograft failure, cardiovascular events, or mortality,^13^ the association between the creatinine-to-cystatin C ratio and mortality.^14^ Nevertheless, to our knowledge, no study has investigated the clinical impact of a low eGFR_cys_/eGFR_cr_ ratio or its determinants relating to body composition. Cardiovascular complications are a major cause of morbidity and remain a leading cause of mortality among KTRs.^15^

The aim of this study was therefore 2-fold as follows: (i) to investigate the association between a decreased eGFR_cys_/eGFR_cr_ ratio and MACEs, all-cause mortality, and allograft loss; and (ii) to identify the clinical and body composition data associated with such a low eGFR_cys_/eGFR_cr_ ratio.

## Methods

### Study Design

This single-center, retrospective, observational study was conducted at Henri Mondor University Hospital, Créteil, France. Informed consent was obtained from all patients, and the study was approved by the institutional ethics committee (Comité de Protection des Personnes Sud Méditerranée – 2023-A02590-45).

All consecutive adults who underwent KT between January 2018 and November 2021, were screened for inclusion. The only exclusion criterion was an unavailable creatinine or cystatin C measurement at 3 months posttransplantation, regardless of the cause. Data collection ended in November 2022. The research was conducted in accordance with the Declaration of Helsinki, and clinical and research activities being reported are consistent with the Principles of the Declaration of Istanbul as outlined in the “Declaration of Istanbul on Organ Trafficking and Transplant Tourism.”

### Variables

KTR characteristics, medical and transplantation history, including immunological and surgical data, as well as kidney donor features were collected from local database and medical records. Donors were categorized as expanded criteria donors if they met the following criteria: aged > 60 years or > 50 years associated with at least 2 conditions among high blood pressure history, serum creatinine > 132 μmol/l, and death from cerebrovascular cause.

Kidney allograft loss was defined as a decline in kidney function requiring long-term kidney replacement therapy. MACEs included acute myocardial infarction, acute heart failure requiring hospitalization with a baseline eGFR_cr_ (CKD-EPI) > 20 ml/min per 1.73 m^2^ (at the time of the event), stroke, cardiovascular death, acute lower limb ischemia, and chronic limb-threatening ischemia. Only MACEs occurring after the eGFR_cys_/eGFR_cr_ ratio assessment were considered.

### Biological Parameters and Assay Methods

Since January 2018, cystatin C has been routinely collected at 3 months posttransplantation in our center, in order to obtain a more accurate assessment of GFR using equations based on creatinine and cystatin C (eGFR_cr-cys_). The 3-month time point after KT was chosen to allow for early assessment of GFR, once potential posttransplant complications have been resolved, and kidney function has stabilized. Plasma creatinine was assessed using the compensated-kinetic Jaffe colorimetric assay CREJ2 (ref. 06407137) until February 2020 and the CREP2 enzymatic assay, thereafter (ref. 05168589). Both assays were used on a Cobas 501 analyzer (Roche Diagnostics). For serum cystatin C measurement, Generation 2 turbidimetric immunoassay CYSC (Ref. 06600239) was performed on a Cobas 501 analyzer (Roche Diagnostics). eGFR_cr_ was determined using the CKD-EPI_cr2009_ and EKFC_cr_ equations.^16^^,^^17^ eGFR_cys_ was calculated using CKD-EPI_cys_ and EKFC_cys_ equations.^18^^,^^19^ eGFR_cr-cys_ was determined using CKD-EPI_cr-cys-2012_^18^ and EKFC_cr-cys_.^19^ The CKD-EPI_cr2009_ and the CKD-EPI_cr-cys-2012_ equations were used rather than the CKD-EPI equations published in 2021, because European medical societies recommend against the adoption of these equations in clinical practice in Europe.^20^^,^^21^ Indeed, the CKD-EPI_cr2021_ equation is less accurate than CKD-EPI_cr2009_ in European patients,^22^ including European KTRs.^23^

Accuracy within 30% (i.e., percentage of eGFR within 30% of the measured GFR) being the conventional criterion used to assess the performance of eGFR equations, a decreased eGFR_cys_/eGFR_cr_ ratio was defined as a ratio < 0.7.^24^ eGFR_cys_/eGFR_cr_ according to CKD-EPI and EKFC equations were hereafter referred to as CKD-EPI_cys_/CKD-EPI_cr_ and EKFC_cys_/EKFC_cr,_ respectively.

### CT Scan Parameters and Analysis

All available abdominal CT scans, performed between 1 year before and 2 weeks after transplantation were analyzed (*N* = 275). If a patient underwent multiple imaging studies, only the one closest to transplantation was considered. CT scan slice thicknesses were of 1.25 mm in 245 patients, > 1.25 mm in 11 patients (maximum: 4 mm), and < 1.25 mm in 19 patients (minimum: 0.5 mm). CT tube voltages were 120 kVp in 222 patients, 100 kVp in 47 patients, and 80 kVp in 6 patients. Unenhanced axial CT sections through the middle of the third lumbar vertebra were used to provide surrogates for skeletal muscle mass and muscle quality, as well as the amount of visceral and subcutaneous fat.^25^^,^^26^ The sum of the areas of the external and internal obliques, paraspinal, rectus abdominis, transversus abdominis, and psoas muscles defined the cross-sectional muscle area, and was measured using semiautomated CT segmentation, after thresholding between −29 and 150 Hounsfield units (HU). The SMI (in cm^2^/m^2^), a surrogate for total body skeletal muscle mass, was obtained by dividing the cross-sectional muscle area, by square height. Muscle density (in HU), a surrogate for myosteatosis, was assessed by using the mean CT attenuation of cross-sectional muscle area at the third lumbar vertebra. Subcutaneous and visceral adipose tissue indexes (cm^2^/m^2^) were determined by segmenting fat outside and inside the abdominal muscular wall respectively, after thresholding according to their specific ranges of CT attenuations: −190 to −30 HU for subcutaneous fat, and −150 to −50 HU for visceral fat. The total adipose tissue index was the sum of subcutaneous and visceral adipose tissue indexes.

### Study End Points

Primary end points were all-cause mortality, MACEs after KT and death-censored kidney allograft loss. Secondary end points consisted of determining clinical and radiological determinants associated with a reduced eGFR_cys_/eGFR_cr_ ratio.

### Statistical Analysis

Categorical data were expressed as numbers and percentages, and continuous variables were expressed as medians with interquartile ranges [IQR]. No continuous variable had a normal distribution. Continuous variables were compared between 2 groups using Mann-Whitney test. Categorical variables were compared using Chi-square test, or Fisher exact test, as appropriate. A logistic regression analysis was used to determine the baseline factors associated with a low eGFR_cys_/eGFR_cr_ ratio (< 0.7). The factors identified as significant in the univariable analysis (with a *P*-value threshold of 0.2) were included in a multivariable model, which was then subjected to stepwise backward elimination, with an exclusion threshold of 5%, until the final model was obtained. Results were expressed as crude odds ratio (e.g., in univariable analysis) and adjusted odds ratio (aOR) (e.g., in multivariable analysis) with 95% CI. Survival curves were generated for patient and allograft survivals, and MACE-free survival by the Kaplan-Meier method, with log-rank tests used for comparisons, according to eGFR_cys_/eGFR_cr_ ratio. Cox proportional hazard univariable and multivariable models were constructed to identify predictive factors of MACEs and mortality. All potential predictive variables yielding associations with a *P*-value < 0.2 in univariable analysis were included as covariates in the multivariable model, to which a backward conditional selection procedure, with a 5% exclusion threshold, was applied until the final model was obtained. Results were expressed as crude hazard ratio (e.g., in univariable analysis) and aHR (e.g., in multivariable analysis) with 95% CI. We excluded highly correlated variables to avoid multicollinearity effects. Two-tailed *P*-values < 0.05 were considered statistically significant. All analyses and graphics were conducted on Stata v15.0 (StataCorp, College Station, TX).

## Results

### Study Sample

A total of 443 patients underwent KT between January 2018, and November 2021, of whom 58 did not have cystatin C measurement at 3 months posttransplant, including 13 patients who died before 3 months, and 21 kidney transplant losses. The baseline characteristics of the 385 included patients are presented in Table 1. The median age was 55.2 years (IQR: 43.8–66.4), most of them being males (64.9%). Body mass index at transplantation was 24.9 (IQR: 21.8–28.1) kg/m^2^. Of the patients, 25.2% had diabetes mellitus. All patients received induction immunosuppressive therapy. Maintenance immunosuppressive therapy relied on a triple association with tacrolimus and steroids in most cases (95.6% and 99.7%, respectively), together with mycophenolic acid (70.9%) or everolimus (28.6%) as the third most common immunosuppressive drug. Of the patients, 275 out of 385 had a CT scan. CT scans were performed between 12 months and 3 months before KT in 62 patients (22.5%, median interval = −7.7 months, IQR = −9.7 to −6.1 months), between 3 months and the day of transplantation in 99 patients (36%), or within 14 days after transplantation in 112 patients (40.7%). The characteristics of these patients with available CT scan are shown in Supplementary Table S1. Patients with a CT scan were more often male than those with no CT scan, and more often had diabetes mellitus or a history of cardiovascular disease before KT. Their eGFR was also slightly lower. Median SMI was 48.2 cm^2^/m^2^ (IQR: 41.4–55); 51.7 cm^2^/m^2^ (IQR: 45.8–57) in males and 41.4 cm^2^/m^2^ (IQR: 35.4–46.4) in females. Muscle density was 35.4 HU (IQR: 28.7–41.3); 36.7 HU (IQR: 30.2–42.7) in males and 32.7 HU (IQR 25–36.2) in females (Table 2). Among these 275 patients, 42 underwent preemptive KT. Their muscle mass and muscle CT attenuation were not statistically different from those of patients on dialysis before KT. Females with preemptive KT had less subcutaneous and visceral fat (Supplementary Table S2).

**Table 1:** Patient characteristics according to their eGFR_cys_/eGFR_cr_ ratio, using the CKD-EPI equations

Variables	Whole cohort	eGFRcys/eGFRcr ⩾ 0.7	eGFRcys/eGFRcr < 0.7	*P*-value
	*N* = 385	*n* = 279	*n* = 106	
Recipient characteristics				
Age, yrs^a^	55.2 (43.8–66.4)	53.7 (42.8–65.6)	58.5 (46.2–70.2)	0.03
Male	250 (64.9%)	174 (62.4%)	76 (71.7%)	0.09
Weight at transplantation (kg)^a^ for males/females	74.8 (65.9–85.3) / 65.5 (57–74)	75.2 (66–85.3) / 65.5 (57.8–74)	73.5 (65–85) / 65.3 (55.6–74)	0.99/0.63
Height (cm)^a^ for men/women	174 (170–179) / 162 (157–168)	175 (170–180) / 163 (158–168)	172.5 (169–177.5) / 160 (157–163)	0.15/0.10
BMI at transplantation (kg/m^2^)^a^	24.9 (21.8–28.1)	24.8 (21.8–27.9)	25.1 (22–28.7)	0.366
Serum creatinine at 3 mo (μmol/l)^a^ for males/females	146 (115–177) / 117 (90–148)	151 (121–186) / 120 (102–151)	123.5 (105–163) / 90 (80–120)	0.0004/0.004
Serum cystatin C at 3 mo (mg/l)^a^ for males/females	1.74 (1.47–2.14) / 1.69 (1.33–2)	1.66 (1.36–1.98) / 1.69 (1.32–1.96)	2.03 (1.64–2.49) / 1.77 (1.48–2.19)	< 0.0001/0.108
CKD-EPI eGFRcr (ml/min per 1.73 m^2^)^a^	46.5 (35.1–62.6)	44.9 (33.9–58.6)	55.3 (37.9–70.8)	0.0002
CKD-EPI eGFRcys (ml/min per 1.73 m^2^)^a^	37.6 (28.4–48.8)	40.5 (30.5–53.9)	31.8 (22.5–42.9)	< 0.0001
CKD-EPIcr-cys-2012 (ml/min per 1.73 m^2^)^a^	41.15 (30.9–54.1)	41.29 (31.5–55.0)	39.93 (28.5–53.8)	0.38
CKD-EPIcr-cys-2012 eGFR categories (< 30; 30–44; 45–59; > 60 ml/min per 1.73 m^2^)	80 (20.8%); 154 (40%), 96 (24.9%), 55 (14.3%)	53 (19%), 119 (42.7%), 64 (22.9%), 43 (15.4%)	24 (22.6%), 34 (32.1%), 34 (32.1%), 14 (13.2%)	0.12
EKFCcr (ml/min per 1.73 m^2^)^a^	46.5 (34.9–61.1)	44.5 (33.7–57.2)	54 (36.9–69.4)	0.0004
EKFCcys (ml/min per 1.73 m^2^)^a^	41.9 (32.9–53.3)	44.6 (35.1–55.7)	36.2 (27.5–47.6)	< 0.0001
EKFCcr-cys (ml/min per 1.73 m^2^)^a^	45.3 (33.9–57.5)	44.7 (35.1–56.8)	46.3 (32.3–59.0)	0.85
EKFCcr-cys eGFR categories (< 30; 30–44; 45–59; > 60 ml/min per 1.73 m^2^)	57 (14.8%), 134 (34.8%), 109 (28.3%), 85 (22.1%)	36 (12.9%), 105 (37.6%), 77 (27.6%), 61 (21.9%)	21 (19.8%), 29 (27.4%), 32 (30.2%), 24 (22.6%)	0.17
Urinary protein-to-creatinine ratio at 3 mo (mg/mmol)^a^	18.1 (9.4–35.7) (*N* = 376)	16.5 (8.4–34.8) (*n* = 274)	20.2 (10.9–41.2) (*n* = 102)	0.015
eGFRcys/eGFRcr < 0.7 by EKFC	54 (14%)	0 (0%)	54 (50.9%)	< 0.0001
Hypertension	354 (92%)	257 (92.1%)	97 (91.5%)	0.85
Diabetes mellitus	97 (25.2%)	59 (21.2%)	38 (35.9%)	0.003
History of cancer	41 (10.7%)	25 (9%)	16 (15.1%)	0.08
History of cardiovascular disease	45 (11.7%)	27 (9.7%)	18 (17%)	0.046
Initial kidney disease				
Diabetic nephropathy	57 (14.8%)	31 (11.1%)	26 (24.5%)	0.0009
Vascular nephropathy	23 (6%)	19 (6.8%)	4 (3.8%)	0.26
Polycystic kidney disease	50 (13%)	38 (13.6%)	12 (11.3%)	0.55
IgA nephropathy	27 (7%)	17 (6.1%)	10 (9.4%)	0.25
Unknown nephropathy	79 (20.5%)	58 (20.8%)	21 (19.8%)	0.83
Other	149 (38.7%)	116 (41.6%)	33 (31.1%)	0.06
Dialysis before KT	331 (86%)	238 (85.3%)	93 (87.7%)	0.54
Donor characteristics				
Age, yrs^a^	58 (47–68)	57 (46–68)	60 (47–71)	0.35
Living kidney donors	64 (16.6%)	53 (19%)	11 (10.4%)	0.042
Expanded criteria donors	185 (48.1%)	129 (46.2%)	56 (52.8%)	0.24
Kidney transplant characteristics				
Cold ischemia, h^a^	14.7 (10.1–19.9)	14.9 (9.2–20.5)	13.9 (11.1–18.6)	0.80
Donor specific antibody at transplantation	164 (42.6%)	119 (42.7%)	45 (42.5%)	0.97
Induction immunosuppressive therapy				
Anti-IL-2 receptor antibodies	137 (35.6%)	94 (33.7%)	43 (40.6%)	0.21
Anti-thymocyte globulin	248 (64.4%)	185 (66.3%)	63 (59.4%)	
Maintenance immunosuppressive therapy				
Ciclosporin	10 (2.6%)	9 (3.2%)	1 (0.9%)	0.21
Tacrolimus	368 (95.6%)	265 (95%)	103 (97.2%)	0.35
Steroids	384 (99.7%)	278 (99.6%)	106 (100%)	0.54
Mycophenolic acid	273 (70.9%)	201 (72%)	72 (67.9%)	0.43
Belatacept	5 (1.3%)	3 (1.1%)	2 (1.9%)	0.53
Everolimus	110 (28.6%)	76 (27.2%)	34 (32.1%)	0.35

BMI, body mass index; CKD-EPI, Chronic Kidney Disease-Epidemiology Collaboration; eGFR, estimated glomerular filtration rate; eGFR_cys_/eGFR_cr_, cystatin C–based to creatinine-based eGFR; EKFC, European Kidney Function Consortium; IL, interleukin; KT, kidney transplantation.

*P*-values were calculated between the eGFRcys/eGFRcr < 0.7 and the eGFRcys/eGFRcr ⩾ 0.7 groups. Continuous variables were compared between groups using a Mann-Whitney test. Categorical variables were compared using Chi-square test, or Fisher exact test, as appropriate. Unless otherwise specified, data are numbers of patients, with percentages in parentheses. *P*-values < 0.05 indicate statistical significance.

a Data are medians, with interquartile ranges in parentheses.

**Table 2:** CT scan morphometric data in kidney transplant recipients according to their eGFR_cys_/eGFR_cr_ ratio, using the CKD-EPI equations

Variables	Whole cohort *N* = 275	eGFRcys/eGFRcr ⩾ 0.7 *n* = 191	eGFRcys/eGFRcr < 0.7 *n* = 84	*P*-value
Subcutaneous adipose tissue index (cm^2^/m^2^)^a^ for total study sample	53.2 (32.6–82.3) (*N* = 275)	53.5 (30.3–82.3) (*n =* 191)	52.9 (35–84.3) (*n =* 84)	0.52
Male/female^a^	49.7 (28.1–70.6) (*N* = 189) / 74.7 (43.7–122.6) (*N* = 86)	49 (26.6–70.6) (*n =* 129) / 74.7 (40.2–122.6) (*n =* 62)	51.2 (33.2–71.7) (*n =* 60) / 71.8 (44.5–115) (*n =* 24)	0.29 / 0.95
Visceral adipose tissue index (cm^2^/m^2^)^a^ for total study sample	50.6 (22.4–76.7) (*N* = 274)	48.5 (20.1–74) (*n =* 190)	61.4 (25.5–81.1) (*n =* 84)	0.10
Male/female^a^	58.6 (27.3–81.8) (*N* = 188) / 41.4 (16.8–69.7) (*N* = 86)	51.9 (22.9–79.5) (*n =* 128) / 45.2 (18.6–65.8) (*n =* 62)	68.4 (35.3–98.1) (*n =* 60) / 27.6 (16–71.5) (*n =* 24)	0.037 / 0.52
Total adipose tissue index (cm^2^/m^2^)^a^ for total study sample	114.1 (61.5–167.5) (*N* = 274)	113.4 (61.9–157.9) (*n =* 196)	117.3 (57.4–184.4) (*n =* 78)	0.68
Male/female^a^	112.3 (58.7–153.1) (*N* = 188) / 119.6 (66.2–187.1) (*N* = 86)	112.6 (60.5–148.6) (*n =* 129) / 114.9 (62.9–187.1) (*n =* 67)	102.3 (52.7–170.7) (*n =* 59) / 134.2 (74.5–190.7) (*n =* 19)	0.73 / 0.63
Skeletal muscle mass index (cm^2^/m^2^)^a^ for total study sample	48.2 (41.4–55) (*N* = 275)	49.8 (42.2–55.8) (*n =* 191)	45.9 (38.3–52.8) (*n =* 84)	0.006
Male/female^a^	51.7 (45.8–57) (*N* = 189) / 41.4 (35.4–46.4) (*N* = 86)	52.8 (47.7–58.2) (*n =* 129) / 41.8 (37.3–47) (*n =* 62)	48 (42.7–54.5) (*n =* 60) / 38 (34.1–45.6) (*n =* 24)	0.001/0.18
Muscle density (HU)^a^ for total study sample	35.4 (28.7–41.3) (*N* = 275)	36.6 (30–42.7) (*n =* 191)	32.2 (25.6–38.7) (*n =* 84)	0.0004
Male/female^a^	36.7 (30.2–42.7) (*N* = 189) / 32.7 (25–36.2) (*N* = 87)	38.3 (32.6–44.1) (*n =* 129) / 31.8 (24.4–35.9) (*n =* 63)	30.5 (25.1–39.3) (*n =* 60) / 33 (27.5–37.9) (*n =* 24)	< 0.0001 / 0.37

CKD-EPI, Chronic Kidney Disease-Epidemiology Collaboration; eGFR, estimated glomerular filtration rate; eGFR_cys_/eGFR_cr_, cystatin C–based to creatinine-based eGFR ratio; HU, Hounsfield units.

*P*-values were calculated between the eGFRcys/eGFRcr < 0.7 and the eGFRcys/eGFRcr ⩾ 0.7 groups.

Continuous variables were compared between groups using a Mann-Whitney test.

a Data are medians, with interquartile ranges in parentheses. *P*-values < 0.05 indicate statistical significance.

### Description of Patients With a Low CKD-EPI_cys_/CKD-EPI_cr_ Ratio

Of the patients, 106 (27.5%) exhibited a CKD-EPI_cys_/CKD-EPI_cr_ < 0.7. These patients were older than those with a normal CKD-EPI_cys_/CKD-EPI_cr_ ratio (58.5 years [IQR: 46.2–70.2] vs. 53.7 years [IQR: 42.8–65.6], *P* = 0.029), had a higher frequency of diabetes mellitus (35.9% vs. 21.2%, *P* = 0.003), and a history of cardiovascular disease before KT (17% vs. 9.7%, *P* = 0.046). They were also less likely to have had a living kidney donor (10.4% vs. 19%, *P* =0.042) (Table 1). Urinary protein-to-creatinine ratio at 3 months was higher in these patients (20.02 mg/mmol [IQR: 10.9–41.2] vs. 16.5 mg/mmol [IQR: 8.4–34.8], *P* = 0.015).

Among the 275 patients with CT scan, 84 patients (30.5%) had a CKD-EPI_cys_/CKD-EPI_cr_ < 0.7 (Table 2). Their SMI was lower than that of patients with a normal ratio (45.9 cm^2^/m^2^ [IQR: 38.3–52.8] vs. 49.8 cm^2^/m^2^ [IQR: 42.2–55.8], *P* = 0.006) as was muscle density (32.2 HU [IQR: 25.6–38.7] vs. 36.6 HU [IQR: 30–42.7], *P* = 0.0004). Except for a higher visceral fat mass in males with low eGFR_cys_/eGFR_cr_ ratio (68.4 cm^2^/m^2^ [IQR: 35.3–98.1] vs. 51.9 cm^2^/m^2^ [IQR: 22.9–79.5], *P* = 0.037), there was no difference in the amount of fat mass between patients with low and normal eGFR_cys_/eGFR_cr_ ratio.

### Description of Patients With a Low EKFC_cys_/EKFC_cr_ Ratio

Fifty-four patients (14%) exhibited an EKFC_cys_/EKFC_cr_ < 0.7 (Supplementary Table S3), with a higher prevalence of male patients in this group compared with patients with a normal ratio (85.2% vs. 61.6% *P* = 0.0008). All patients with an EKFC_cys_/EKFC_cr_ < 0.7 had a low CKD-EPI ratio. History of diabetes mellitus and diabetic nephropathy were more frequent (44.4% vs. 22.1%, *P* = 0.0004; and 25.9% vs. 13%, *P* = 0.013, respectively); however, history of cardiovascular disease before KT was not more frequent than in patients with a normal EKFC_cys_/EKFC_cr_ ratio. The urinary protein-to-creatinine ratio at 3 months was higher in these patients (22.1 mg/mmol [IQR: 13.1–41.5] vs. 16.9 mg/mmol [8.8–34.9], *P* = 0.033). Muscle density was lower in this low EKFC_cys_/EKFC_cr_ ratio group (32 HU [IQR: 23.7–37.7] vs. 36 HU [IQR: 29.4–42], *P* = 0.007), but SMI was similar (Supplementary Table S4). There was no difference in the amount of fat mass between patients with low versus normal EKFC_cys_/EKFC_cr_ ratio. Finally, no difference was observed regarding donor and kidney transplant characteristics.

### Primary End Points

During the median follow-up time of 24 months (IQR: 14–37), 42 patients (10.9 %) died, 17 experienced allograft loss (4.4 %), and 39 experienced MACEs (10.1 %), with median times of 14 months (IQR: 6–28), 18 months (IQR: 9–30) and 12 months (IQR: 2–24), respectively.

**Figure 1:**
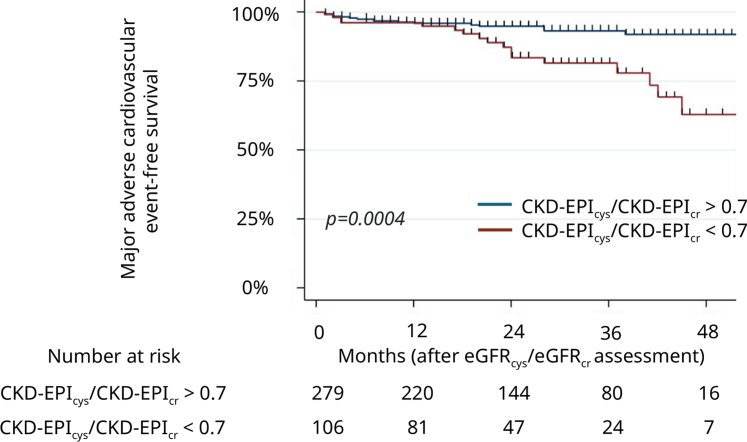
Kaplan-Meier curves for major adverse cardiovascular–free survival in patients with normal CKD-EPI_cys_/CKD-EPI_cr_ ratio and low CKD-EPI_cys_/CKD-EPI_cr_ ratio (< 0.7). The *P*-value was determined by a log-rank test. CKD-EPI, Chronic Kidney Disease-Epidemiology Collaboration; CKD-EPI_cys_/CKD-EPI_cr_ ratio, cystatin C–based to creatinine-based eGFR ratio using CKD-EPI equations; eGFR, estimated glomerular filtration rate.

A low 3-month CKD-EPI_cys_/CKD-EPI_cr_ ratio was independently associated with MACEs after KT with an aHR of 2.46 (95% CI: 1.24–4.87, *P* = 0.01), as was a low EKFC_cys_/EKFC_cr_ ratio (aHR: 2.29, 95% CI: 1.11–4.72, *P* = 0.025) (Table 3, Supplementary Table S5, Figure 1, and Supplementary Figure S1). Other conditions associated with MACEs were age (aHR: 1.04, 95% CI: 1.01–1.07, *P* = 0.017), and diabetes mellitus (aHR: 2.60, 95% CI: 1.29–5.23, *P* = 0.007). The univariable analyses for MACEs are presented in Supplementary Table S5.

**Table 3:** Univariable and multivariable Cox proportional hazards regression for major adverse cardiovascular events in kidney transplant recipients

Variables	Univariable analysis			Multivariable analysis 1			Multivariable analysis 2		
	cHR	95% CI	*P*-value	aHR	95% CI	*P*-value	aHR	95% CI	*P*-value
Age (for 1 yr increase)	1.05	1.02–1.08	0.0005	1.04	1.01–1.07	0.017	1.04	1.01–1.07	0.009
Diabetes mellitus	3.77	1.90–7.49	0.0001	2.60	1.29–5.23	0.007	2.59	1.28–5.23	0.008
History of cardiovascular disease	2.13	0.93–4.90	0.075	-	-	-	-	-	-
CKD-EPIcr-cys-2012	0.98	0.96–1.00	0.078	-	-	-	-	-	-
EKFCcr-cys	0.98	0.96–1.00	0.078	-	-	-	-	-	-
eGFRcys/eGFRcr < 0.7 using EKFC equations	2.78	1.35–5.71	0.005	-	-	-	2.29	1.11–4.72	0.025
eGFRcys/eGFRcr < 0.7 using CKD-EPI equations	3.16	1.61–6.19	0.0008	2.46	1.24–4.87	0.01	-	-	-
Deceased kidney donor	2.36	0.72–7.73	0.16	-	-	-	-	-	-

aHR, adjusted hazard ratio; cHR, crude hazard ratio; CKD-EPI, Chronic Kidney Disease-Epidemiology Collaboration; CI, confidence interval; eGFR, estimated glomerular filtration rate; eGFR_cys_/eGFR_cr_, cystatin C–based to creatinine-based eGFR ratio; EKFC, European Kidney Function Consortium.

*P*-values were calculated using a univariable then a multivariable Cox proportional hazards regression analysis. Backward conditional selection procedure, with a 5% exclusion threshold, was applied until the final model was obtained. In multivariable analysis 1, variables were adjusted for age, diabetes mellitus status and eGFRcys/eGFRcr < 0.7 using CKD-EPI equations. In multivariable analysis 2, variables were adjusted for age, diabetes mellitus status and eGFRcys/eGFRcr < 0.7 using EKFC equations. *P*-values < 0.05 indicate statistical significance. No variable was forced in this multivariable model. *P*-values < 0.05 indicate statistical significance.

**Figure 2:**
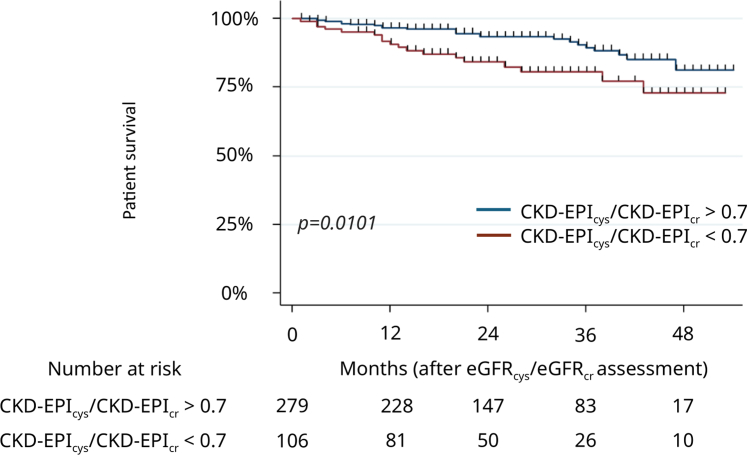
Kaplan-Meier curves for patient survival in patients with normal CKD-EPI_cys_/CKD-EPI_cr_ ratio and low CKD-EPI_cys_/CKD-EPI_cr_ ratio (< 0.7). The *P*-value was determined by a log-rank test. CKD-EPI, Chronic Kidney Disease-Epidemiology Collaboration; CKD-EPI_cys_/CKD-EPI_cr_ ratio, cystatin C–based to creatinine-based eGFR ratio using CKD-EPI equations; eGFR, estimated glomerular filtration rate.

A CKD-EPI_cys_/CKD-EPI_cr_ < 0.7 was associated with all-cause mortality in univariable analysis (Table 4, Supplementary Table S6, and Figure 2), but not in multivariable analysis, in which only age was a factor associated with mortality (aHR: 1.08, 95% CI: 1.05–1.12, *P* = 0.000) (Table 4). EKFC_cys_/EKFC_cr_ < 0.7 was not a risk factor for mortality either in univariable or multivariable analysis (Table 4, Supplementary Table S6, and Supplementary Figure S2).

**Table 4:** Univariable and multivariable Cox proportional hazards regression for mortality in kidney transplant recipients

Variables	Univariable analysis			Multivariable analysis		
	cHR	95% CI	*P*-value	aHR	95% CI	*P*-value
Age (for 1 yr increase)	1.09	1.06–1.12	< 0.0001	1.08	1.05–1.12	< 0.0001
CKD-EPIcr-cys-2012	0.96	0.94–0.99	0.001	-	-	-
EKFCcr-cys	0.96	0.94–0.98	< 0.0001	-	-	-
eGFRcys/eGFRcr < 0.7 using CKD-EPI equations	2.20	1.19–4.08	0.012	1.48	0.78–2.81	0.23
Diabetes mellitus	2.10	1.13–3.92	0.019	-	-	-
History of cancer	2.22	1.06–4.65	0.035	-	-	-
Dialysis before kidney transplantation	2.37	0.73–7.68	0.150	2.02	0.62–6.64	0.25
Deceased kidney donor	4.42	1.07–18.31	0.041	-	-	-

aHR, adjusted hazard ratio; cHR, crude hazard ratio; CI, confidence interval; CI, confidence interval; CKD-EPI, Chronic Kidney Disease-Epidemiology Collaboration; eGFR, estimated glomerular filtration rate; eGFR_cys_/eGFR_cr_, cystatin C–based to creatinine-based eGFR ratio; EKFC, European Kidney Function Consortium.

*P*-values were calculated using a univariable then a multivariable Cox proportional hazards regression analysis. Backward conditional selection procedure, with a 5% exclusion threshold, was applied until the final model was obtained. In multivariable analysis, variables were adjusted for age, eGFRcys/eGFRcr < 0.7 using CKD-EPI equations, and dialysis before kidney transplantation. No variable was forced in this multivariable model. P-values < 0.05 indicate statistical significance.

**Figure 3:**
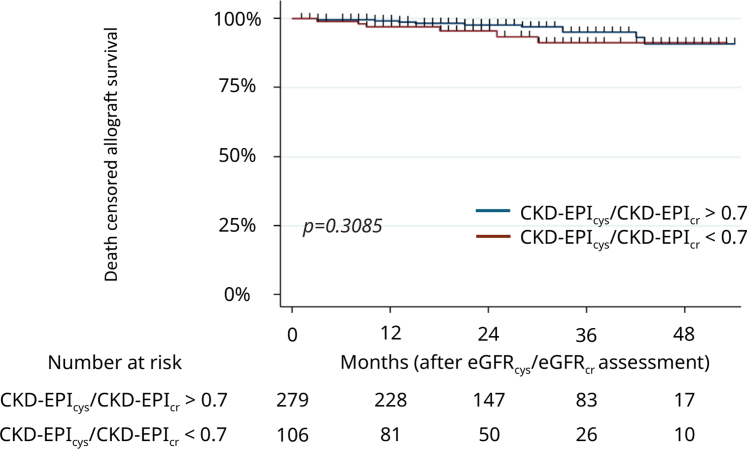
Kaplan-Meier curves for death-censored allograft survival in patients with normal CKD-EPI_cys_/CKD-EPI_cr_ ratio and low CKD-EPI_cys_/CKD-EPI_cr_ ratio (< 0.7). The *P*-value was determined by a log-rank test. CKD-EPI, Chronic Kidney Disease-Epidemiology Collaboration; CKD-EPI_cys_/CKD-EPI_cr_ ratio, cystatin C–based to creatinine-based eGFR ratio using CKD-EPI equations; eGFR, estimated glomerular filtration rate.

A low eGFR_cys_/eGFR_cr_ ratio was not associated with allograft loss occurrence (Figure 3, Supplementary Figure S3). Cold ischemia was the only factor associated with allograft loss in univariable analysis (HR: 1.07, 95% CI: 1.0–1.15, *P* = 0.042) (Supplementary Table S7), and no variable was associated with allograft loss in multivariable analysis.

### Secondary End Points

The only factor associated with a CKD-EPI_cys_/CKD-EPI_cr_ < 0.7 in the entire cohort was diabetes mellitus, with an aOR of 2.02 (95% CI: 1.23–3.32, *P* = 0.006) (Table 5 and Supplementary Table S8). In the subgroup of patients with an available CT scan, variables associated with a low CKD-EPI_cys_/CKD-EPI_cr_ ratio were male sex (aOR: 2.60, 95% CI: 1.32–5.10, *P* = 0.006), muscle density (aOR: 0.95, 95% CI: 0.92–0.98, *P* = 0.001), and SMI (aOR: 0.95, 95% CI: 0.92–0.98, *P* = 0.003).

**Table 5:** Association between patient characteristics and a eGFR_cys_/eGFR_cr_ ratio < 0.7, using the CKD-EPI equations in univariable and multivariable logistic regression analysis

Variables	Univariable analysis			Multivariable analysis		
	cOR	95% CI	*P*-value	aOR	95% CI	*P*-value
Whole study sample (*N* = 385)						
Age (for 1 yr increase)	1.02	1.00–1.03	0.029	-	-	-
Male	1.53	0.94–2.49	0.087	-	-	-
Deceased kidney donor	2.03	1.01–4.05	0.042	1.88	0.93–3.79	0.078
History of cancer	1.81	0.92–3.54	0.081	1.93	0.97–3.83	0.062
Diabetes mellitus	2.08	1.28–3.40	0.003	2.02	1.23–3.32	0.006
History of cardiovascular disease	1.91	1.00–3.63	0.046	-	-	-
CKD-EPIcr-cys-2012	0.99	0.98–1.01	0.32	-	-	-

Variables	Univariable analysis			Multivariable analysis		
	cOR	95% CI	*P*-value	aOR	95% CI	*P*-value
KT recipients with CT scan available (*n* = 275)						
Male	1.20	0.68–2.11	0.52	2.6	1.32–5.10	0.006
CKD-EPIcr-cys-2012	1.00	0.99–1.02	0.98	-	-	-
Diabetes mellitus	2.00	1.15–3.47	0.013	-	-	-
Muscle density (for 1 HU increase)	0.94	0.92–0.97	0.0004	0.95	0.92–0.98	0.001
Skeletal muscle mass index (for 1 cm^2^/m^2^ increase)	0.96	0.93–0.99	0.006	0.95	0.92–0.98	0.003
Visceral adipose tissue index (for 1 cm^2^/m^2^ increase)	1.01	1.00–1.01	0.10	-	-	-

aOR, adjusted odds ratio; cOR, crude odds ratio; CI, confidence interval; CKD-EPI, Chronic Kidney Disease-Epidemiology Collaboration; CT, computed tomography; eGFR, estimated glomerular filtration rate; eGFR_cys_/eGFR_cr_ cystatin C–based to creatinine-based eGFR ratio; HU, Hounsfield units; KT, kidney transplantation.

*P*-values were calculated using a univariable then a multivariable logistic regression analysis. Backward conditional selection procedure, with a 5% exclusion threshold, was applied until the final model was obtained. In multivariable analysis with the whole study sample (*N* = 385), variables were adjusted for deceased kidney donor, history of cancer and diabetes mellitus status. In multivariable analysis with CT scan available (*n* = 275), variables were adjusted for sex category, muscle density and skeletal muscle mass index. Because body composition is highly sex-dependent, the sex variable was forced in models with CT scan available (*n* = 275). *P*-values < 0.05 indicate statistical significance.

Determining factors of an EKFC_cys_/EKFC_cr_ ratio < 0.7 in the whole study sample were male sex (aOR: 3.5, 95% CI: 1.59–7.71, *P* = 0.002) and diabetes mellitus (aOR: 2.76, 95% CI: 1.50–5.07, *P* = 0.001) (Supplementary Tables S9 and S10). In the subgroup of patients with CT scan, variables associated with a low EKFC_cys_/EKFC_cr_ ratio were the male sex (aOR: 4.39, 95% CI: 1.68–11.52, *P* = 0.003), diabetes mellitus (aOR: 2.14, 95% CI: 1.03–4.49, *P* = 0.043), and muscle density (aOR: 0.94, 95% CI: 0.9–0.98, *P* = 0.003).

## Discussion

In the present study analyzing for the first time the significance of low eGFR_cys_/eGFR_cr_ in KTRs, we show that such a low ratio 3 months after KT was independently associated with MACEs, regardless of whether the CKD-EPI or the EKFC equations were used. All-cause mortality more than 3 months after transplantation was associated with a low CKD-EPI_cys_/CKD-EPI_cr_ ratio in univariable analysis but not in multivariable analysis. A low eGFR_cys_/eGFR_cr_ ratio was not associated with kidney transplant loss. The sole factor associated with a low CKD-EPI_cys_/CKD-EPI_cr_ ratio was diabetes mellitus. In the subgroup of patients with available CT scan, male sex, myosteatosis, and low muscle mass were associated with this low ratio. A decreased EKFC_cys_/EKFC_cr_ ratio was much less prevalent than a decreased CKD-EPI_cys_/CKD-EPI_cr_ ratio; was associated with male sex and diabetes mellitus in the whole cohort; and with male sex, diabetes mellitus, and myosteatosis in the subgroup of patients with an available CT scan (no association with muscle mass).

Recent data highlight that a low eGFR_cys_/eGFR_cr_ ratio is associated with an increase in cardiovascular complications,^7^ including heart failure, as well as risk of acute kidney injury, end-stage kidney disease, and mortality, both in hypertensive population^7^, ^8^, ^9^ and CKD population,^10^^,^^27^ as well as in the general population.^28^ The association we found between the incidence of MACEs and low eGFR_cys_/eGFR_cr_ is in line with the study of Keddis *et al.*^13^ which showed that eGFR_cys_ outperforms eGFR_cr_ in predicting adverse outcomes in KTRs, including cardiovascular events, allograft loss, and mortality; and that these results were primarily due to non-GFR determinants of cystatin C. The association of a decreased CKD-EPI_cys_/CKD-EPI_cr_ ratio with mortality, which we found only in the univariable analysis could be due to a lack of statistical power due to the low number of events. In a study including 1592 KTRs, Choi *et al.* found that a low creatinine-to-cystatin C ratio was associated with an increased risk of death.^14^ Another possible explanation could be that the CKD-EPI equations model the GFR with small age-dependent biases. Indeed, Delanaye *et al.* recently showed that the differences between eGFR_cys_ and eGFR_cr_ were dependent on age for the CKD-EPI equations, but not for the EKFC equations.^29^ This age-independent EKFC-based eGFR ratio was not associated with mortality in our study, even in univariable analysis.

Finally, we found no evidence to suggest that the low eGFR_cys_/eGFR_cr_ ratio would be a risk factor for allograft loss, but again the number of events was small and the follow-up period may not have been long enough to establish such an association.

It should be noted that whereas the eGFR_cys_/eGFR_cr_ ratio was associated with the occurrence of MACEs, eGFR alone was not. This therefore indicates that the occurrence of MACEs in KTRs is not a consequence of poor kidney function, but rather the result of a combination of non-GFR determinants of creatinine and cystatin C. Indeed, a low eGFR_cys_/eGFR_cr_ ratio can be attributable to several non-GFR determinants of endogenous serum concentration of these biomarkers. First, serum creatinine may be abnormally low because of low muscle mass. As expected, myopenia (i.e., a low SMI) was associated with a low CKD-EPI_cys_/CKD-EPI_cr_ ratio. Reduced protein intake can also lead to abnormally low creatinine levels,^30^ but we were unable to assess this effect, because we do not conduct dietary surveys as part of routine care, nor do we collect 24-hour urine analyses to measure urine urea nitrogen. Finally, a high level of tubular creatinine secretion may lead to a reduced eGFR_cys_/eGFR_cr_ ratio; this feature was not assessable either because we did not perform GFR measurement with exogenous tracers.^31^

Decreased muscle density, which was associated with low eGFR_cys_/eGFR_cr_ ratio, is indicative of myosteatosis, that is, fat deposition within muscles.^32^, ^33^, ^34^ Myosteatosis has been associated with reduced muscle strength and function,^35^ insulin resistance,^36^ hypertension,^37^ coronary calcifications,^38^ poor outcomes in various oncological patients,^39^, ^40^, ^41^ as well as in KTRs.^42^ To the best of our knowledge, it has never been demonstrated that myosteatosis is responsible for a reduction in creatinine generation. The alternative hypothesis of an association between myosteatosis and abnormally high serum concentrations of cystatin C is plausible. Indeed, the causes of myosteatosis mainly include age,^43^ physical inactivity,^44^ insulin resistance,^36^^,^^45^ and inflammation.^46^ Insulin resistance and inflammation have also been identified as non-GFR determinants of cystatin C^47^ although this remains debatable to date.^48^^,^^49^

Although previously published data indicate that visceral and subcutaneous adiposity was associated with abnormally high values of cystatin C,^50^ we did not find such an association in our study.

Diabetes mellitus was found to be independently associated with a decreased eGFR_cys_/eGFR_cr_ ratio, regardless of which equation was used. Whether the diabetic status of patients would be a non-GFR determinant of cystatin C is still unclear. In a cross-sectional study of 1139 KTRs who underwent iothalamate urinary clearance, diabetes was associated with an eGFR_cys_ that underestimated the measured GFR.^12^ In another analysis of 3418 patients with measured GFR and cystatin C measurement, Stevens *et al.*^47^ found that diabetes was a non-GFR-related determinant of serum cystatin C. However, Delanaye *et al.*^48^ found, in a study involving 6158 patients with measured GFR and cystatin C measurement that eGFR_cr_ and eGFR_cys_ had the same performance in diabetic and nondiabetic patients. The impact of systemic corticosteroid therapy, a recognized non-GFR determinant of serum cystatin C, could not be assessed, as all but one of the patients received this treatment. However, the dose administered at 3 months after KT was 5 mg/d, so the impact should have been negligible.^51^^,^^52^ Thyroid-stimulating hormone values were not analyzed either.

A hypothesis increasingly put forward to explain the discrepancies between serum creatinine and cystatin C values is the shrunken pore syndrome,^53^ also called selective glomerular hypofiltration syndrome. Reduction in the size and elongation of the glomerular fenestrated endothelial cells could result in decreased excretion of proteins between 5 and 30 kDa, including not only cystatin C, but also cytokines and inflammatory proteins.^54^ This may provide a rationale for the increased cardiovascular risk and mortality observed in patients with low eGFR_cys_/eGFR_cr_ ratio.^28^^,^^55^ Our study did not allow us to assess whether KTRs with a low eGFR_cys_/eGFR_cr_ ratio actually had a selective glomerular hypofiltration syndrome.

In addition to being attributable to non-GFR determinants of creatinine and cystatin C, a low eGFR_cys_/eGFR_cr_ ratio can be explained by the mathematical construction of eGFR equations. This is demonstrated in our study by the higher proportion of patients with a low ratio when using CKD-EPI equations than when using EKFC equations, with all patients with a low EKFC ratio also having a low CKD-EPI ratio. In a recent study of more than 15,000 patients, Delanaye et al. also found a higher proportion of patients with discrepancies between eGFR_cys_ and eGFR_cr_ when using CKD-EPI equations compared with EKFC equations.^29^ It is interesting to note that the discrepancies observed with the EKFC equations were balanced (i.e., equivalent proportions of eGFR_cys_ < eGFR_cr_ and eGFR_cr_ < eGFR_cys_), whereas for the CKD-EPI equations, eGFR_cys_ < eGFR_cr_ was by far the most common situation (3 times more frequent than eGFR_cys_ > eGFR_cr_).

The fact that almost all patients during the inclusion period had a simultaneous creatinine and cystatin C assay 3 months after transplantation (385/409 living patients with a functional renal allograft) is a strength of the study. The same is true of the novelty of studying the relationship between body composition and the eGFR_cys_/eGFR_cr_ ratio in KTRs. However, our study has several limitations in addition to its single-center retrospective design. Because cystatin C measurement is not routinely performed in our center until 3 months after KT, we could not analyze whether the KTRs who died or who lost the allograft very early, or who had a very early MACEs, also had a decreased eGFR_cys_/eGFR_cr_ ratio. We also had no dynamic data on the evolution of the eGFRcys/eGFRcr ratio over time and were therefore unable to test whether earlier or later assessments of this ratio would provide more accurate prognostic data. The relatively short duration of follow-up resulted in a small number of events for several variables of interest, including death and allograft loss. Over 25% of the study sample did not have a CT scan available, therefore a selection bias affecting the results derived from the CT scan cannot be ruled out. Some data that could lead to discordance between creatinine and cystatin C could not be analyzed; these include protein intake and tubular secretion of creatinine, and smoking, thyroid function, and shrunken pore syndrome for cystatin C.

In conclusion, our study highlights that in KTRs, a low eGFR_cys_/eGFR_cr_ ratio is associated with the occurrence of MACEs. Our other main finding was that body composition abnormalities, such as low muscle mass and myosteatosis, but not high adiposity, could be key factors of such a low ratio. These results provide support for the widespread use of cystatin C, not only to improve the accuracy of GFR estimation using creatinine- and cystatin C–based equations, but also to identify KTRs at risk of adverse clinical events. If these results are confirmed in the future, other questions will need to be resolved, such as the potential reversibility of low eGFR_cys_/eGFR_cr_ through the improvement of the physical conditions and metabolic profiles of KTRs, and the therapeutic approaches to be implemented to mitigate the cardiovascular risk highlighted by this discrepancy between eGFR_cys_ and eGFR_cr_.

## Disclosure

All the authors declared no competing interests.

## Supplementary Material

Supplementary File (PDF) Figure S1. Kaplan-Meier curves for major adverse cardiovascular–free survival in patients with normal EKFC_cys_/EKFC_cr_ ratio and low EKFC_cys_/EKFC_cr_ ratio (< 0.7). Figure S2. Kaplan-Meier curves for patient survival in patients with normal EKFC_cys_/EKFC_cr_ ratio and low EKFC_cys_/EKFC_cr_ ratio (< 0.7). Figure S3. Kaplan-Meier curves for death-censored allograft survival in patients with normal EKFC_cys_/EKFC_cr_ ratio and low EKFC_cys_/EKFC_cr_ ratio (< 0.7). Table S1. Patient characteristics according to their CT scan prior to transplant status. Table S2. CT-based morphometric characteristics of patients and their clinical features, depending on whether kidney transplantation was preemptive or performed after dialysis treatment. Table S3. Patient characteristics according to their eGFR_cys_/eGFR_cr_ ratio, using the EKFC equations. Table S4. CT scan morphometric data in kidney transplant recipients according to their eGFR_cys_/eGFR_cr_ ratio, using the EKFC equations. Table S5. Univariable Cox proportional hazards regression for major adverse cardiovascular events in kidney transplant recipients. Table S6. Univariable Cox proportional hazards regression for mortality events in kidney transplant recipients. Table S7. Univariable Cox proportional hazards regression for allograft loss in kidney transplant recipients. Table S8. Association between patient characteristics and an eGFRcys/eGFRcr ratio < 0.7, using the CKD-EPI equation in univariable analysis. Table S9. Association between patient characteristics and an eGFR_cys_/eGFR_cr_ ratio < 0.7, using the EKFC equation in univariable and multivariable logistic regression analysis. Table S10. Association between patient characteristics and an eGFRcys/eGFRcr ratio < 0.7, using the EKFC equation in univariable analysis. STROBE checklist.
