# Supplementary material for: Low Cystatin C–to–Creatinine Estimated GFR Ratio Predicts Post–Kidney Transplant Cardiovascular Events
Source: Kidney Int Rep. 2025 Aug 26;10(11):3905–17. doi: 10.1016/j.ekir.2025.08.029 (PMC12640023; doi:10.1016/j.ekir.2025.08.029)
Supplement: Supplementary File (PDF) — Figure S1. Kaplan-Meier curves for major adverse cardiovascular–free survival in patients with normal EKFCcys/EKFCcr ratio and low EKFCcys/EKFCcr ratio (< 0.7). Figure S2. Kaplan-Meier curves for patient survival in patients with normal EKFCcys/EKFCcr ratio and low EKFCcys/EKFCcr ratio (< 0.7). Figure S3. Kaplan-Meier curves for death-censored allograft survival in patients with normal EKFCcys/EKFCcr ratio and low EKFCcys/EKFCcr ratio (< 0.7). Table S1. Patient characteristics according to their CT scan prior to transplant status. Table S2. CT-based morphometric characteristics of patients and their clinical features, depending on whether kidney transplantation was preemptive or performed after dialysis treatment. Table S3. Patient characteristics according to their eGFRcys/eGFRcr ratio, using the EKFC equations. Table S4. CT scan morphometric data in kidney transplant recipients according to their eGFRcys/eGFRcr ratio, using the EKFC equations. Table S5. Univariable Cox proportional hazards regression for major adverse cardiovascular events in kidney transplant recipients. Table S6. Univariable Cox proportional hazards regression for mortality events in kidney transplant recipients. Table S7. Univariable Cox proportional hazards regression for allograft loss in kidney transplant recipients. Table S8. Association between patient characteristics and an eGFRcys/eGFRcr ratio < 0.7, using the CKD-EPI equation in univariable analysis. Table S9. Association between patient characteristics and an eGFRcys/eGFRcr ratio < 0.7, using the EKFC equation in univariable and multivariable logistic regression analysis. Table S10. Association between patient characteristics and an eGFRcys/eGFRcr ratio < 0.7, using the EKFC equation in univariable analysis. STROBE checklist. [file mmc1.pdf]

Supplementary Material

Supplementary figures

Figure S1: Kaplan-Meier curves for major adverse cardiovascular-free survival in patients with normal $EKFC_{cys}/EKFC_{cr}$ ratio and low $EKFC_{cys}/EKFC_{cr}$ ratio (< 0.7).

Fig. S1

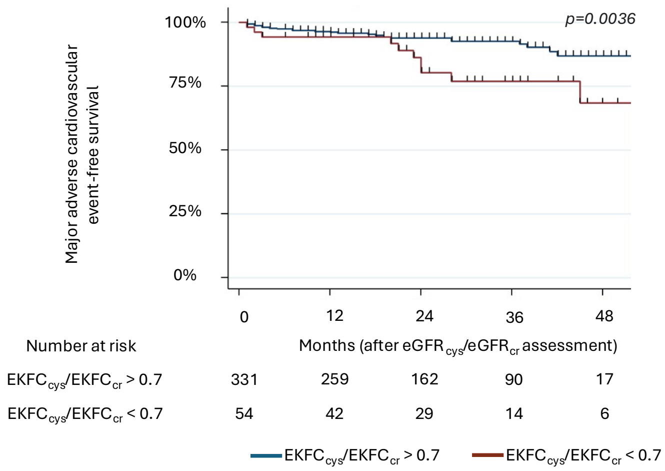

The P-value was determined by a log-rank test.

Figure S2: Kaplan-Meier curves for patient survival in patients with normal $EKFC_{cys}/EKFC_{cr}$ ratio and low $EKFC_{cys}/EKFC_{cr}$ ratio (< 0.7).

Fig. S2

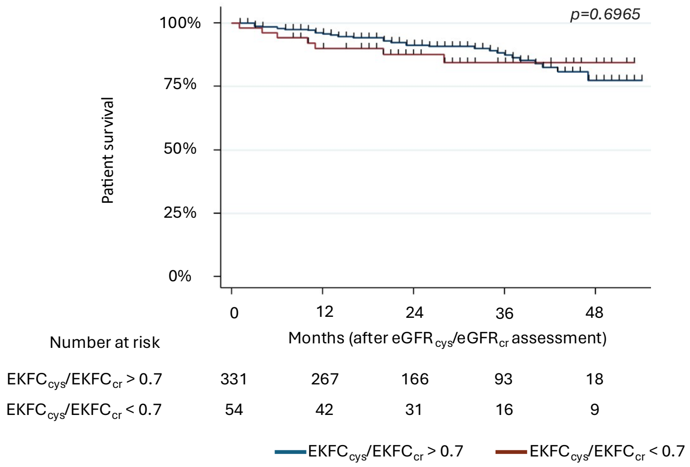

The P-value was determined by a log-rank test.

Figure S3: Kaplan-Meier curves for Death censored allograft survival in patients with normal $EKFC_{cys}/EKFC_{cr}$ ratio and low $EKFC_{cys}/EKFC_{cr}$ ratio (< 0.7).

Fig. S3

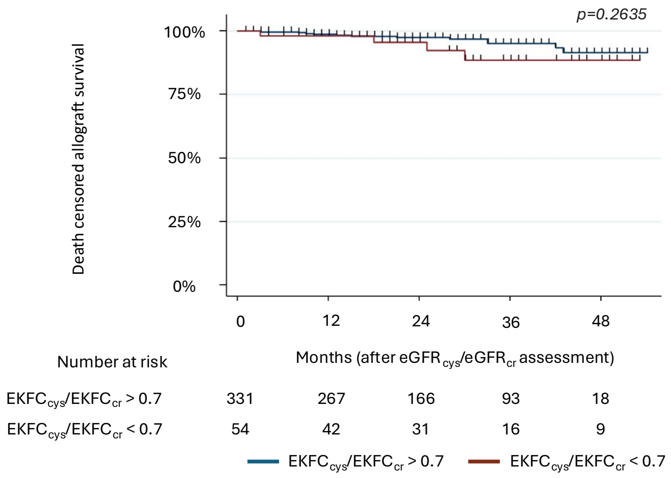

The P-value was determined by a log-rank test.

Supplementary Tables

Table S1. Patient characteristics according to their CT scan prior to transplant status

Variables	Whole cohort<br><br>N=385	No CT scan in the year prior to transplant<br><br>N=110	CT scan in the year prior to transplant<br><br>N=275	p-value
**Recipient characteristics**				
Age, years*	55.2 (43.8;66.4)	51.7 (42.8;63.7)	56.7 (44.9;67.8)	0.07
Male	250 (64.9%)	61 (55.5%)	189 (68.7%)	**0.014**
Weight at transplantation (kg)* for males/females	74.8 (65.9;85.3) / 65.5 (57;74)	79.8 (68;88) / 67.4 (59.9;74.1)	73 (65;83.8) / 65 (55.5;74)	0.084/0.51
Height (cm)* for males/females	174 (170;179) / 162 (157;168)	176 (170;182) / 163 (160;167)	173 (170;178) / 161 (156;168)	0.15/0.24
BMI at transplantation (kg/m2)*	24.9 (21.8;28.1)	25.2 (22.6;28.1)	24.7 (21.8;28.1)	0.41
Serum creatinine at 3 months (µmol/L)* for males/females	146 (115;177) / 117 (90;148)	127 (111;157) / 120 (99;145)	151 (118;186) / 115.5 (87;150)	**0.004**/0.38
Serum cystatin C at 3 months (mg/L)* for males/females	1.74 (1.47;2.14) / 1.69 (1.33;2.00)	1.58 (1.34;1.81) / 1.62 (1.36;1.96)	1.84 (1.52;2.23) / 1.70 (1.33;2.03)	**0.0009**/0.85
CKD-EPI eGFRcr (mL/min/1.73m2)*	46.5 (35.1;62.6)	49.9 (37.3;67.2)	45.2 (33.4;61)	**0.046**
CKD-EPI eGFRcys (mL/min/1.73m2)*	37.6 (28.4;48.8)	42.3 (30;56.2)	35.4 (27;48)	**0.008**
CKD-EPIcr-cys-2012 (mL/min/1.73m2)*	41.2 (30.90;54.07)	44.6 (31.98;58.89)	39.4 (30.11;52.73)	**0.018**
EKFCcr (mL/min/1.73m2)*	46.5 (34.9;61.1)	50.1 (36.9;66)	44.5 (33.4;59.2)	**0.039**
EKFCcys (mL/min/1.73m2)*	41.9 (32.9;53.3)	46 (35.4;59.9)	40.7 (31.7;52.2)	**0.004**
EKFCcr-cys (mL/min/1.73m2)*	45.33 (33.88;57.53)	48.05 (35.87;62.17)	43.70 (33.46;56.26)	**0.014**
Urinary protein-creatinine ratio at 3 months (mg/mmol)*	18.05 (9.41;35.70) (*N*=376)	15.76 (7.80;40.06) (*N*=107)	18.65 (10;34.95) (*N*=269)	0.21
eGFRcys/eGFRcr < 0.7 by CKD-EPI	106 (27.5%)	22 (20%)	84 (30.6%)	**0.036**
eGFRcys/eGFRcr < 0.7 by EKFC	54 (14%)	13 (11.8%)	41 (14.9%)	0.43
Hypertension	354 (92%)	96 (87.3%)	258 (93.8%)	**0.033**
Diabetes mellitus	97 (25.2%)	20 (18.2%)	77 (28%)	**0.045**
History of cancer	41 (10.7%)	9 (8.2%)	32 (11.6%)	0.32
History of cardiovascular disease	45 (11.7%)	5 (4.6%)	40 (14.6%)	**0.006**
Initial kidney disease				
Diabetic nephropathy	57 (14.8%)	9 (8.2%)	48 (17.5%)	**0.021**
Vascular nephropathy	23 (6%)	7 (6.4%)	16 (5.8%)	0.84
Polycystic kidney disease	50 (13%)	20 (18.2%)	30 (10.9%)	0.055
IgA nephropathy	27 (7%)	7 (6.4%)	20 (7.3%)	0.75
Unknown nephropathy	79 (20.5%)	23 (20.9%)	56 (20.4%)	0.91
Other	149 (38.7%)	44 (40%)	105 (38.2%)	0.74
Dialysis before kidney transplantation	331 (86%)	98 (89.1%)	233 (84.7%)	0.27
**Donor characteristics**				
Age, years*	58 (47;68)	55 (44;65)	59 (47;70)	**0.041**
Living kidney donors	64 (16.6%)	22 (20%)	42 (15.3%)	0.26
Expanded criteria donors	185 (48.1%)	43 (39.1%)	142 (51.6%)	**0.026**
**Kidney transplant characteristics**				
Cold ischemia, hours*	14.7 (10.1;19.9)	13.6 (7.8;19.2)	15.2 (11.1;20.1)	**0.032**
Donor specific antibody at transplantation	164 (42.6%)	46 (41.8%)	118 (42.9%)	0.85
Induction immunosuppressive therapy				
Anti-IL-2 receptor antibodies	137 (35.6%)	38 (34.6%)	99 (36%)	0.79
Anti-thymocyte globulin	248 (64.4%)	72 (65.5%)	176 (64%)	
Maintenance immunosuppressive therapy				
Ciclosporin	10 (2.6%)	5 (4.6%)	5 (1.8%)	0.13
Tacrolimus	368 (95.6%)	102 (92.7%)	266 (96.7%)	0.084

Steroids	384 (99.7%)	110 (100%)	274 (99.6%)	0.53
Mycophenolic acid	273 (70.9%)	80 (72.7%)	193 (70.2%)	0.62
Belatacept	5 (1.3%)	2 (1.8%)	3 (1.1%)	0.57
Everolimus	110 (28.6%)	30 (27.3%)	80 (29.1%)	0.72

P-values were calculated between the "No CT scan in the year prior to transplant" and "CT scan in the year prior to transplant" groups. Continuous variables were compared between groups a Mann-Whitney test. Categorical variables were compared using Chi-square test, or Fisher exact test, as appropriate.
Unless otherwise specified, data are numbers of patients, with percentages in parentheses. * Data are medians, with IQRs in parentheses. P-values < 0.05 are in bold.

Table S2. CT-based morphometric characteristics of patients and their clinical features, depending on whether kidney transplantation was pre-emptive or performed after dialysis treatment.

**Variables**	**CT scan in the year prior to transplant** N=275	**Preemptive kidney transplantation** N=42	**Non-preemptive kidney transplantation** N=233	**p-value**
**Recipient characteristics**				
Age, years*	56.7 (44.9;67.8)	55.6 (47.5; 64.8)	57.5 (44.3;68.0)	0.74
Male	189 (68.7%)	29 (69.1%)	160 (68.7%)	0.48
Weight at transplantation (kg)* for males/females	73 (65;83.8) / 65 (55.5;74)	74 (66.2; 87) / 59 (53; 64)	73 (64.8; 83) / 68 (56;74)	0.49/0.11
Height (cm)* for males/females	173 (170;178) / 161 (156;168)	176 (172;181) / 160 (153;165)	173 (170;178) / 161 (157;168)	0.13/0.58
BMI at transplantation (kg/m2)*	24.7 (21.8;28.1)	23.9 (21.5;28.2)	24.8 (21.8;28.1)	0.31
CKD-EPIcr-cys-2012 (mL/min/1.73m2)*	39.4 (30.1;52.7)	45.1 (35.8;54.3)	37.8 (30.3;51.9)	0.067
EKFCcr-cys (mL/min/1.73m2)*	43.7 (33.5;56.3)	49.8 (38.9;60.2)	41.2 (32.8;54.2)	**0.037**
eGFRcys/eGFRcr < 0.7 by CKD-EPI	84 (30.6%)	12 (28.6%)	72 (30.9%)	0.86
eGFRcys/eGFRcr < 0.7 by EKFC	41 (14.9%)	8 (19.1%)	33 (14.2%)	0.48
Hypertension	258 (93.8%)	38 (90.5%)	220 (94.4%)	0.31
Diabetes mellitus	77 (28%)	12 (28.6%)	65 (27.9%)	1
History of cancer	32 (11.6%)	5 (11.9%)	27 (11.6%)	1
History of cardiovascular disease	40 (14.6%)	8 (19.1%)	32 (13.7%)	0.35
Subcutaneous adipose tissue index (cm2/m2)* for total study sample	53.2 (32.6;82.3) (N=275)	38.1 (27.4;78.9) (N=42)	54.9 (34.1;83.1) (N=233)	**0.019**
Male/female*	49.7 (28.1;70.6) (N=189) / 74.7 (43.7;122.6) (N=86)	35.7 (23.7;56.7) (N=29) / 44.2 (37.3;83.3) (N=13)	51.6 (29.6;70.8) (N=160) / 83.2 (46;125.7) (N=73)	0.13/**0.025**
Visceral adipose tissue index (cm2/m2)* for total study sample	50.6 (22.4;76.7) (N=274)	41.7 (13.6;74) (N=42)	53.5 (24.7;76.9) (N=232)	0.12
Male/female*	58.6 (27.3;81.8) (N=188) / 41.4 (16.8;69.7) (N=86)	54 (23.3;78.9) (N=29) / 13.6 (10.7;35.3) (N=13)	60 (27.5 ;82) (N=159) / 45.1 (20.3;69.8) (N=73)	0.58/**0.013**
Skeletal muscle mass index (cm2/m2)* for total study sample	48.2 (41.4;55) (N=275)	47.4 (41.9;52.9) (N=42)	48.3 (41.4;55.4) (N=233)	0.65
Male/female*	51.7 (45.8;57) (N=189) / 41.4 (35.4;46.4) (N=86)	51.2 (45.2;55.8) (N=29) / 40.8 (35.7;44) (N=13)	52 (45.8;57.2) (N=160) / 41.6 (35.4;46.9) (N=73)	0.74/0.58
Muscle density (HU)* for total study sample	35.4 (28.7;41.3) (N=275)	36.9 (31.8;42.8) (N=42)	35 (28.6;40.4) (N=233)	0.13
Male/female*	36.7 (30.2;42.7) (N=189) / 32.7 (25;36.2) (N=87)	39.2 (32.1;43.1) (N=29) / 32.8 (31.8;35.9) (N=13)	36.6 (30;42.3) (N=160) / 31.8 (24;36.2) (N=74)	0.30/0.23

P-values were calculated between the "Preemptive kidney transplantation" and "Non-preemptive kidney transplantation" groups. Continuous variables were compared between groups a Mann-Whitney test. Categorical variables were compared using Chi-square test, or Fisher exact test, as appropriate.
Unless otherwise specified, data are numbers of patients, with percentages in parentheses. * Data are medians, with IQRs in parentheses. P-values < 0.05 are in bold.

Table S3. Patient characteristics according to their $eGFR_{cys}/eGFR_{cr}$ ratio, using the EKFC equations.

Variables	Whole cohort<br>*N*=385	eGFRcys/eGFRcr ⩾ 0,7<br>*N*=331	eGFRcys/eGFRcr < 0,7<br>*N*=54	p-value
**Recipient characteristics**				
Age, years*	55.2 (43.8;66.4)	55.2 (42.8;66.2)	57.2 (49.5;67.8)	0.20
Male	250 (64.9%)	204 (61.6%)	46 (85.2%)	**0.0008**
Weight at transplantation (kg)* for males/females	74.8 (65.9;85.3) / 65.5 (57;74)	75 (66;84.6) / 65.5 (57.1;74)	74.3 (65.1;89) / 63.2 (45.5;79.2)	0.82/0.70
Height (cm)* for males/females	174 (170;179) / 162 (157;168)	174.5 (170;180) / 163 (158;168)	172.5 (169;177) / 158.5 (156.5;160)	0.26/0.15
BMI at transplantation (kg/m2)*	24.9 (21.8;28.1)	24.8 (21.9;27.9)	26.5 (21.7;29)	0.40
Serum creatinine at 3 months (µmol/L)* for males/females	146 (115;177) / 117 (90;148)	150 (120;182.5) / 117 (94;148)	117.5 (98;162) / 79 (70.5;132.5)	**0.0002/0.048**
Serum cystatin C at 3 months (mg/L)* for males/females	1.74 (1.47;2.14) / 1.69 (1.33;2)	1.68 (1.41;2.05) / 1.69 (1.33;1.98)	2.03 (1.67;2.71) / 1.91 (1.48;2.93)	**<0.0001**/0.21
CKD-EPI eGFRcr (mL/min/1.73m2)*	46.5 (35.1;62.6)	45.3 (34.6;59.9)	61.1 (39.9;77.7)	**0.0004**
CKD-EPI eGFRcys (mL/min/1.73m2)*	37.6 (28.4;48.8)	39 (29.6;50.8)	30.6 (20.3;42.2)	**<0.0001**
CKD-EPIcr-cys-2012 (mL/min/1.73m2)*	41.2 (30.90;54.07)	41.2 (31.15;53.92)	40.7 (27.22;55.36)	0.65
CKD-EPIcr-cys-2012 eGFR categories (< 30; 30-44; 45-59; > 60 ml/min/1.73m2)	80 (20.8%); 154 (40%), 96 (24.9%), 55 (14.3%)	64 (19.3%), 142 (42.9%), 77 (23.3%), 48 (14.5%)	16 (29.6%), 12 (22.2%), 19 (35.2%), 7 (13%)	0.059
EKFCcr (mL/min/1.73m2)*	46.5 (34.9;61.1)	45 (34;58.4)	58.8 (41.1;76)	**<0.0004**
EKFCcys (mL/min/1.73m2)*	41.9 (32.9;53.3)	43.1 (34.4;54.9)	34.2 (24.1;45.8)	**<0.0001**
EKFCcr-cys (mL/min/1.73m2)*	45.3 (33.9;57.5)	44.9 (34.1;56.9)	46.89 (32.1;61.0)	0.79
EKFCcr-cys eGFR categories (< 30; 30-44; 45-59; > 60 ml/min/1.73m2)	57 (14.8%), 134 (34.8%), 109 (28.3%), 85 (22.1%)	46 (13.9%), 120 (36.3%), 95 (28.7%), 70 (21.1%)	11 (20.4%), 14 (25.9%), 14 (25.9%), 15 (27.8%)	0.27
Urinary protein-creatinine ratio at 3 months (mg/mmol)*	18.1 (9.4;35.7) (*N*=376)	16.9 (8.8;34.9) (*N*=324)	22.1 (13.1;41.5) (*N*=52)	**0.033**
eGFRcys/eGFRcr < 0.7 by CKD-EPI	106 (27.5%)	52 (15.7%)	54 (100%)	**<0.0001**
Hypertension	354 (92%)	303 (91.5%)	51 (94.4%)	0.47
Diabetes mellitus	97 (25.2%)	73 (22.1%)	24 (44.4%)	**0.0004**
History of cancer	41 (10.7%)	33 (10%)	8 (14.8%)	0.28
History of cardiovascular disease	45 (11.7%)	35 (10.6%)	10 (18.5%)	0.09
Initial kidney disease				
Diabetic nephropathy	57 (14.8%)	43 (13%)	14 (25.9%)	**0.013**
Vascular nephropathy	23 (6%)	21 (6.3%)	2 (3.7%)	0.45
Polycystic kidney disease	50 (13%)	43 (13%)	7 (13%)	0.99
IgA nephropathy	27 (7%)	22 (6.7%)	5 (9.3%)	0.49
Unknown nephropathy	79 (20.5%)	69 (20.9%)	10 (18.5%)	0.69
Other	149 (38.7%)	133 (40.2%)	16 (29.6%)	0.14
Dialysis before kidney transplantation	331 (86%)	286 (86.4%)	45 (83.3%)	0.55
**Donor characteristics**				
Age, years*	58 (47;68)	57 (46;68)	62 (48;70)	0.15
Living kidney donors	64 (16.6%)	58 (17.5%)	6 (11.1%)	0.24
Expanded criteria donors	185 (48.1%)	153 (46.2%)	32 (59.3%)	0.06
**Kidney transplant characteristics**				
Cold ischemia, hours*	14.7 (10.1;19.9)	14.7 (9.8;19.9)	14.6 (12;19.4)	0.50
Donor specific antibody at transplantation	164 (42.6%)	139 (42%)	25 (46.3%)	0.55
Induction immunosuppressive				
Anti-IL-2 receptor antibodies	137 (35.6%)	115 (34.7%)	22 (40.7%)	0.39
Anti-thymocyte globulin	248 (64.4%)	216 (65.3%)	32 (59.3%)	
Maintenance immunosuppressive therapy				
Ciclosporin	10 (2.6%)	9 (2.7%)	1 (1.9%)	0.71
Tacrolimus	368 (95.6%)	316 (95.5%)	52 (96.3%)	0.78
Steroids	384 (99.7%)	330 (99.7%)	54 (100%)	0.69
Mycophenolic acid	273 (70.9%)	237 (71.6%)	36 (66.7%)	0.46
Belatacept	5 (1.3%)	5 (1.5%)	0 (0%)	0.36
Everolimus	110 (28.6%)	92 (27.8%)	18 (33.3%)	0.40

P-values were calculated between the eGFRcys/eGFRcr < 0,7 and the eGFRcys/eGFRcr ⩾ 0,7 groups. Continuous variables were compared between groups using a Mann-Whitney test. Categorical variables were compared using Chi-square test, or Fisher exact test, as appropriate. Unless otherwise specified, data are numbers of patients, with percentages in parentheses. * Data are medians, with IQRs in parentheses. P-values < 0.05 are in bold.

Table S4. CT scan morphometric data in kidney transplant recipients according to their $eGFR_{cys}/eGFR_{cr}$ ratio, using the EKFC equations.

**Variables**	**Whole cohort** N = 275	**eGFRcys/eGFRcr ⩾ 0,7** N = 234	**eGFRcys/eGFRcr < 0,7** N = 41	**p-value**
**Subcutaneous adipose tissue index** (cm2/m2)* for total study sample	53.2 (32.6;82.3) (N=275)	53.1 (32;83.2) (N=234)	53.2 (34.9;74.8) (N=41)	0.96
Male/female*	49.7 (28.1;70.6) (N=189) / 74.7 (43.7;122.6) (N=86)	49.2 (26.9;68.4) (N=154) / 79.9 (44.2;123.7) (N=80)	53.6 (34.9;74.8) (N=35) / 45.5 (32.5;98.3) (N=6)	0.19/0.44
**Visceral adipose tissue index** (cm2/m2)* for total study sample	50.6 (22.4;76.7) (N=274)	49.7 (20.3;75.5) (N=233)	68.3 (25.9;82) (N=41)	0.19
Male/female*	58.6 (27.3;81.8) (N=188) / 41.4 (16.8;69.7) (N=86)	54.2 (25.2;79.5) (N=153) / 44.1 (17.4;69.7) (N=80)	72.2 (34.3;96.1) (N=35) / 25.5 (15.5;29.2) (N=6)	0.14/0.22
**Total adipose tissue index** (cm2/m2)* for total study sample	114.1 (61.5;167.5) (*N*=274)	114 (62.5;160.5) (*N*=232)	117.7 (52.1;184.6) (*N*=42)	0.86
Male/female*	112.3 (58.7;153.1) (N=188) / 119.6 (66.2;187.1) (N=86)	112.3 (61;148.7) (N=154) / 119.6 (66.2;186.3) (N=78)	110 (42.3;184.4) (N=34) / 140.4 (79.4;226.1) (N=8)	0.93/0.50
**Skeletal muscle mass index** (cm2/m2)* for total study sample	48.2 (41.4;55) (N=275)	48.9 (41.6;55.4) (N=234)	46.9 (40.4;53.8) (N=41)	0.33
Male/female*	51.7 (45.8;57) (N=189) / 41.4 (35.4;46.4) (N=86)	52.1 (46.7;57.6) (N=154) / 41.6 (36.1;46.4) (N=80)	48 (42.9;55) (N=35) / 35.4 (34.6;44.8) (N=6)	**0.030**/0.23
**Muscle density** (HU)* for total study sample	35.4 (28.7;41.3) (N=275)	36 (29.4;42) (N=234)	32 (23.7;37.7) (N=41)	**0.007**
Male/female*	36.7 (30.2;42.7) (N=189) / 32.7 (25;36.2) (N=87)	37.5 (31.7;43.8) (N=154) / 32.3 (25;36.2) (N=81)	31.6 (23.5;39.5) (N=35) / 34.1 (27.4;35.1) (N=6)	**0.0004**/0.82

P-values were calculated between the eGFRcys/eGFRcr < 0,7 and the eGFRcys/eGFRcr ⩾ 0,7 groups.
Continuous variables were compared between groups using a Mann-Whitney test.* Data are medians, with IQRs in parentheses. P-values < 0.05 are in bold.

Table S5. Univariable Cox proportional hazards regression for major adverse cardiovascular events in kidney transplant recipients.

Variables	N=377		
	**N**	**HR (CI95%)**	**p-value**
Age (for 1 year increase)	377	1.05 (1.02;1.08)	**<0.0001**
Weight at transplantation	377	1.01 (0.98;1.03)	0.623
Height	377	0.99 (0.96;1.03)	0.635
BMI at transplantation	377	1.04 (0.96;1.13)	0.367
Male	244	0.74 (0.37;1.46)	0.382
Hypertension	346	1.57 (0.37;6.57)	0.538
Diabetes mellitus	92	3.77 (1.90;7.49)	**<0.0001**
History of cancer	41	1.70 (0.70;4.12)	0.241
History of cardiovascular disease	42	2.13 (0.93;4.90)	0.075
Dialysis before kidney transplantation	324	1.39 (0.49;3.95)	0.535
eGFRcys/eGFRcr < 0.7 by EKFC at 3 months	53	2.78 (1.35;5.71)	**0.005**
eGFRcys/eGFRcr < 0.7 by CKD-EPI at 3 months	104	3.16 (1.61;6.19)	**0.001**
Urinary protein-creatinine ratio at 3 months	368	1.00 (1.00;1.01)	0.163
CKD-EPIcr-cys-2012 at 3 months	377	0.98 (0.96;1.00)	0.078
EKFCcr-cys at 3 months	377	0.98 (0.96;1.00)	0.078
Deceased kidney donor	317	2.36 (0.72;7.73)	0.157
Expanded criteria donor	183	2.77 (1.32;5.79)	**0.007**
Donor sex	203	0.97 (0.49;1.90)	0.923
Donor age	377	1.04 (1.01;1.06)	**0.003**
Cold ischemia	377	1.02 (0.98;1.07)	0.339
Skeletal muscle mass index (for 1 cm2/m2 increase)	269	0.97 (0.93;1.00)	0.083
Muscle CT attenuation (for 1 HU increase)	269	0.94 (0.90;0.97)	**0.001**
Subcutaneous adipose tissue index (for 1 cm2/m2 increase)	269	1.00 (0.99;1.01)	0.427
Visceral adipose tissue index (for 1 cm2/m2 increase)	268	1.01 (1.00;1.02)	0.086
Total adipose tissue index (for 1 cm2/m2 increase)	269	1.00 (0.99;1.01)	0.976
Donor specific antibody at transplantation	161	1.42 (0.72;2.78)	0.308
Induction immunosuppressive therapy with anti-thymocyte globulin	244	1.20 (0.58;2.46)	0.62
**Maintenance immunosuppressive therapy**			
Mycophenolic acid	268	1.04 (0.50;2.18)	0.917
Certican	107	1.00 (0.48;2.09)	0.997
**Initial kidney disease**			
Diabetic nephropathy	53	3.68 (1.81;7.48)	**<0.0001**
Vascular nephropathy	23	0.46 (0.06;3.39)	0.447
Polycystic kidney disease	49	0.88 (0.31;2.51)	0.816
IgA nephropathy	27	0.37 (0.05;2.73)	0.331
Unknown nephropathy	79	0.46 (0.16;1.30)	0.142
Other	146	0.90 (0.45;1.83)	0.777

P-values < 0.05 are in bold.

Table S6. Univariable Cox proportional hazards regression for mortality events in kidney transplant recipients.

Variables	N=382		
	N	HR (CI95%)	p-value
Age (for 1 year increase)	382	1.09 (1.06;1.12)	**<0.0001**
Weight at transplantation	382	1.00 (0.98;1.02)	0.956
Height	382	0.98 (0.95;1.01)	0.214
BMI at transplantation	382	1.03 (0.96;1.11)	0.37
Male	247	1.28 (0.65;2.52)	0.467
Hypertension	351	1.23 (0.38;3.99)	0.733
Diabetes mellitus	96	2.10 (1.13;3.92)	**0.019**
History of cancer	41	2.22 (1.06;4.65)	**0.035**
History of cardiovascular disease	44	1.28 (0.54;3.05)	0.578
Dialysis before kidney transplantation	329	2.37 (0.73;7.68)	0.15
eGFRcys/eGFRcr < 0.7 by EKFC at 3 months	53	1.18 (0.52;2.66)	0.697
eGFRcys/eGFRcr < 0.7 by CKD-EPI at 3 months	104	2.20 (1.19;4.08)	**0.012**
Urinary protein-creatinine ratio at 3 months	373	1.01 (1.00;1.01)	**0.006**
CKD-EPIcr-cys-2012 at 3 months	382	0.96 (0.94;0.99)	**0.001**
EKFCcr-cys at 3 months	382	0.96 (0.94;0.98)	**<0.0001**
Deceased kidney donor	320	4.42 (1.07;18.31)	**0.041**
Expanded criteria donor	185	4.72 (2.18;10.23)	**<0.0001**
Donor sex	207	1.33 (0.71;2.48)	0.371
Donor age	382	1.06 (1.03;1.08)	**<0.0001**
Cold ischemia	382	1.02 (0.98;1.06)	0.372
Skeletal muscle mass index (for 1 cm2/m2 increase)	273	1.00 (0.97;1.03)	0.981
Muscle CT attenuation (for 1 HU increase)	273	0.93 (0.90;0.97)	**<0.0001**
Subcutaneous adipose tissue index (for 1 cm2/m2 increase)	273	1.00 (0.99;1.01)	0.977
Visceral adipose tissue index (for 1 cm2/m2 increase)	272	1.02 (1.01;1.03)	**<0.0001**
Total adipose tissue index (for 1 cm2/m2 increase)	272	1.00 (0.99;1.00)	0.955
Donor specific antibody at transplantation	163	0.99 (0.53;1.85)	0.977
Induction immunosuppressive therapy with anti-thymocyte globulin	247	0.90 (0.48;1.68)	0.737
**Maintenance immunosuppressive therapy**			
Mycophenolic acid	271	0.84 (0.44;1.61)	0.607
Everolimus	109	1.23 (0.64;2.34)	0.535
**Initial kidney disease**			
Diabetic nephropathy	56	1.94 (0.95;3.97)	0.068
Vascular nephropathy	23	0.38 (0.05;2.79)	0.343
Polycystic kidney disease	49	0.50 (0.15;1.62)	0.248
IgA nephropathy	27	1.05 (0.32;3.39)	0.94
Unknown nephropathy	79	0.90 (0.41;1.94)	0.784
Other	148	1.06 (0.56;1.98)	0.864

P-values < 0.05 are in bold.

Table S7. Univariable Cox proportional hazards regression for allograft loss in kidney transplant recipients.

Variables	N=382		
	N	HR (CI95%)	p-value
Age (for 1 year increase)	382	1.03 (0.99;1.06)	0.176
Weight at transplantation	382	1.01 (0.97;1.04)	0.724
Height	382	1.00 (0.95;1.05)	0.975
BMI at transplantation	382	1.03 (0.91;1.16)	0.634
Male	247	1.60 (0.52;4.98)	0.413
Hypertension	351	1.48 (0.19;11.25)	0.706
Diabetes mellitus	96	1.37 (0.48;3.95)	0.556
History of cancer	41	1.78 (0.50;6.25)	0.371
History of cardiovascular disease	44	2.50 (0.80;7.76)	0.113
Dialysis before kidney transplantation	329	2.83 (0.37;21.46)	0.314
eGFRcys/eGFRcr < 0.7 by EKFC at 3 months	53	1.89 (0.61;5.87)	0.272
eGFRcys/eGFRcr < 0.7 by CKD-EPI at 3 months	104	1.68 (0.61;4.63)	0.314
Urinary protein-creatinine ratio at 3 months	373	1.00 (0.99;1.01)	0.964
CKD-EPIcr-cys-2012 at 3 months	382	0.98 (0.94;1.01)	0.138
EKFCcr-cys at 3 months	382	0.97 (0.94;1.01)	0.108
Deceased kidney donor	320	2,64 (0.40-20.75)	0.083
Expanded criteria donor	207	1.62 (0.59;4.48)	0.352
Donor sex	185	2.58 (0.89;7.42)	0.08
Donor age	382	1.03 (0.99;1.06)	0.105
Cold ischemia	382	1.07 (1.00;1.15)	**0.042**
Skeletal muscle mass index (for 1 cm2/m2 increase)	273	1.01 (0.95;1.08)	0.689
Muscle CT attenuation (for 1 HU increase)	273	1.05 (0.97;1.13)	0.227
Subcutaneous adipose tissue index (for 1 cm2/m2 increase)	273	0.99 (0.97;1.01)	0.226
Visceral adipose tissue index (for 1 cm2/m2 increase)	272	1.01 (0.99;1.02)	0.534
Total adipose tissue index (for 1 cm2/m2 increase)	272	1.00 (0.99;1.00)	0.393
Donor specific antibody at transplantation	163	0.65 (0.22;1.86)	0.418
Induction immunosuppressive therapy with anti-thymocyte globulin	247	0.44 (0.16;1.18)	0.102
**Maintenance immunosuppressive therapy**			
Mycophenolic acid	271	0.55 (0.21;1.49)	0.241
Everolimus	109	1.87 (0.70;5.03)	0.213
**Initial kidney disease**			
Diabetic nephropathy	56	2.00 (0.64;6.20)	0.231
Vascular nephropathy	23	1.05 (0.14;7.94)	0.964
Polycystic kidney disease	49	0.43 (0.06;3.23)	0.41
IgA nephropathy	27	3.04 (0.87;10.67)	0.083
Unknown nephropathy	79	1.22 (0.39;3.78)	0.731
Other	148	0.38 (0.11;1.34)	0.133

P-values < 0.05 are in bold.

Table S8. Association between patient characteristics and an eGFRcys/eGFRcr ratio < 0,7, using the CKD-EPI equation in univariable analysis.

Variables	N=385	eGFRcys/eGFRcr ⩾ 0,7 N=279	eGFRcys/eGFRcr < 0,7 N=106		p-value
	N	N	N	OR (CI95%)	
Age (for 1 year increase)	385	279	106	1.02 (1.00;1.03)	**0.026**
Weight at transplantation	385	279	106	1.01 (0.99;1.02)	0.44
Height	385	279	106	0.99 (0.97;1.02)	0.667
BMI at transplantation	385	279	106	1.03 (0.97;1.08)	0.316
Male	250	174	76	1.53 (0.94;2.49)	0.088
Hypertension	354	257	97	0.92 (0.41;2.07)	0.845
Diabetes mellitus	97	59	38	2.08 (1.28;3.40)	**0.003**
History of cancer	41	25	16	1.81 (0.92;3.54)	0.085
History of cardiovascular disease	45	27	18	1.91 (1.00;3.63)	**0.049**
Dialysis before kidney transplantation	331	238	93	1.23 (0.63;2.40)	0.54
Urinary protein-creatinine ratio at 3 months	376	274	102	1.00 (1.00;1.01)	0.066
CKD-EPIcr-cys-2012 at 3 months	385	279	106	0.99 (0.98;1.01)	0.319
EKFCcr-cys at 3 months	385	279	106	1.00 (0.99;1.01)	0.818
Deceased kidney donor	321	226	95	2.03 (1.01;4.05)	**0.046**
Expanded criteria donor	185	129	56	1.31 (0.84;2.06)	0.238
Donor sex	209	145	64	1.41 (0.89;2.22)	0.14
Donor age	385	279	106	1.00 (0.99;1.02)	0.503
Cold ischemia	385	279	106	1.00 (0.97;1.03)	0.883
Skeletal muscle mass index (for 1 cm2/m2 increase)	275	191	84	0.96 (0.93;0.99)	**0.004**
Muscle CT attenuation (for 1 HU increase)	275	191	84	0.94 (0.92;0.97)	**<0.0001**
Subcutaneous adipose tissue index (for 1 cm2/m2 increase)	275	191	84	1.00 (1.00;1.01)	0.538
Visceral adipose tissue index (for 1 cm2/m2 increase)	274	190	84	1.01 (1.00;1.01)	**0.05**
Total adipose tissue index (for 1 cm2/m2 increase)	274	196	78	1.00 (1.00;1.00)	0.683
CT scan between 12 months and 3 months before kidney transplantation	62	42	20	1.14 (0.62;2.10)	0.753
Donor specific antibody at transplantation	164	119	45	0.99 (0.63;1.56)	0.972
Induction immunosuppressive therapy with anti-thymocyte globulin	248	185	63	0.74 (0.47;1.18)	0.209
**Maintenance immunosuppressive therapy**					
Mycophenolic acid	273	201	72	0.82 (0.51;1.33)	0.427
Everolimus	110	76	34	1.26 (0.78;2.05)	0.349

**Initial kidney disease**					
Diabetic nephropathy	57	31	26	2.60 (1.46;4.64)	**0.001**
Vascular nephropathy	23	19	4	0.54 (0.18;1.62)	0.268
Polycystic kidney disease	50	38	12	0.81 (0.41;1.62)	0.549
IgA nephropathy	27	17	10	1.61 (0.71;3.63)	0.255
Unknown nephropathy	79	58	21	0.94 (0.54;1.65)	0.832
Other	149	116	33	0.64 (0.39;1.02)	0.061

P-values were calculated between the eGFRcys/eGFRcr < 0,7 and the eGFRcys/eGFRcr ⩾ 0,7 groups. P-values < 0.05 are in bold.

Table S9. Association between patient characteristics and a $eGFR_{cys}/eGFR_{cr}$ ratio < 0,7, using the EKFC equation in univariable and multivariable logistic regression analysis.

**Variables**	**Univariable analysis**			**Multivariable analysis**		
	**cOR**	**95% CI**	**p-value**	**aOR**	**95% CI**	**p-value**
**<u>Whole study sample (N=385)</u>**						
Age (for 1 year increase)	1.01	0.99-1.04	0.15	-	-	-
Male	3.58	1.64-7.83	0.001	3.5	1.59-7.71	**0.002**
Diabetes mellitus	2.83	1.56-5.13	0.0006	2.76	1.50-5.07	**0.001**
UPCR (mg/mmol) (N=376)	1	1.00-1.01	0.15	-	-	-
EKFCcr-cys	1	0.99-1.02	0.73	-	-	-

**Variables**	**Univariable analysis**			**Multivariable analysis**		
	**cOR**	**95% CI**	**p-value**	**aOR**	**95% CI**	**p-value**
**<u>KT recipients with CT scan available (N=275)</u>**						
Male	3.03	1.22-7.51	0,013	4.39	1.68-11.52	**0.003**
Diabetes mellitus	2.96	1.50-5.84	0,001	2.14	1.03-4.49	**0.043**
EKFCcr-cys	1.01	0.99-1.03	0.27	-	-	-
Skeletal muscle mass index (for 1 cm2/m2 increase)	0.98	0.95-1.01	0.33	-	-	-
Muscle density (for 1 HU increase)	0.94	0.91-0.98	0.007	0.94	0.90-0.98	**0.003**
Visceral adipose tissue index (for 1 cm2/m2 increase)	1.01	1.00-1.02	0.19	-	-	-
CT scan between 12 months and 3 months before kidney transplantation	0.43	(0.16-1.15)	0.104	-	-	-

aOR, adjusted odds ratio; cOR, crude odds ratio CI, confidence interval. P-values were calculated using a univariable then a multivariable logistic regression analysis. Backward conditional selection procedure, with a 5% exclusion threshold, was applied until the final model was obtained. In multivariable analysis with the whole study sample (N=385), variables were adjusted for sex and diabetes mellitus status. In multivariable analysis with CT scan available (N=275), variables were adjusted for sex category, diabetes mellitus status and muscle density (HU). . Because body composition is highly sex-dependent, the sex variable was forced in models with CT scan available (N=275) .P-values < 0.05 are in bold.

Table S10. Association between patient characteristics and an eGFRcys/eGFRcr ratio < 0.7, using the EKFC equation in univariable analysis.

Variables	N=385	eGFRcys/eGFRcr ⩾ 0,7 N=331	eGFRcys/eGFRcr < 0,7 N=54		p-value
	N	N	N	OR (CI95%)	
Age (for 1 year increase)	385	331	54	1.01 (0.99;1.04)	0.148
Weight at transplantation	385	331	54	1.01 (0.99;1.03)	0.172
Height	385	331	54	1.01 (0.98;1.04)	0.429
BMI at transplantation	385	331	54	1.03 (0.97;1.10)	0.349
Male	250	204	46	3.58 (1.64;7.83)	**0.001**
Hypertension	354	303	51	1.57 (0.46;5.36)	0.471
Diabetes mellitus	97	73	24	2.83 (1.56;5.13)	**0.001**
History of cancer	41	33	8	1.57 (0.68;3.61)	0.288
History of cardiovascular disease	45	35	10	1.92 (0.89;4.15)	0.097
Dialysis before kidney transplantation	331	286	45	0.79 (0.36;1.72)	0.547
Urinary protein-creatinine ratio at 3 months	376	324	52	1.00 (1.00;1.01)	0.15
CKD-EPIcr-cys-2012 at 3 months	385	331	54	1.00 (0.98;1.01)	0.619
EKFCcr-cys at 3 months	385	331	54	1.00 (0.99;1.02)	0.731
Deceased kidney donor	321	273	48	1.70 (0.69;4.16)	0.245
Expanded criteria donor	185	153	32	1.75 (0.97;3.17)	0.063
Donor sex	209	176	33	1.38 (0.77;2.49)	0.279
Donor age	385	331	54	1.01 (0.99;1.03)	0.186
Cold ischemia	385	331	54	1.02 (0.98;1.06)	0.444
Skeletal muscle mass index (for 1 cm2/m2 increase)	275	234	41	0.98 (0.95;1.01)	0.217
Muscle CT attenuation (for 1 HU increase)	275	234	41	0.94 (0.91;0.98)	**0.003**
Subcutaneous adipose tissue index (for 1 cm2/m2 increase)	275	234	41	1.00 (0.99;1.01)	0.697
Visceral adipose tissue index (for 1 cm2/m2 increase)	274	233	41	1.01 (1.00;1.02)	0.076
Total adipose tissue index (for 1 cm2/m2 increase)	274	232	42	1.00 (1.00;1.01)	0.756
Donor specific antibody at transplantation	164	139	25	1.19 (0.67;2.12)	0.554
CT scan between 12 months and 3 months before kidney transplantation	62	57	5	0.43 (0.16;1.15)	0.104
Induction immunosuppressive therapy with anti-thymocyte globulin	248	216	32	0.77 (0.43;1.39)	0.394
**Maintenance immunosuppressive therapy**					
Mycophenolic acid	273	237	36	0.79 (0.43;1.47)	0.46
Everolimus	110	92	18	1.30 (0.70;2.40)	0.404
**Initial kidney disease**					
Diabetic nephropathy	57	43	14	2.34 (1.18;4.66)	**0.015**
Vascular nephropathy	23	21	2	0.57 (0.13;2.49)	0.453
Polycystic kidney disease	50	43	7	1.00 (0.42;2.35)	0.995
IgA nephropathy	27	22	5	1.43 (0.52;3.96)	0.488
Unknown nephropathy	79	69	10	0.86 (0.41;1.80)	0.695
Other	149	133	16	0.63 (0.34;1.17)	0.142

P-values were calculated between the eGFRcys/eGFRcr < 0,7 and the eGFRcys/eGFRcr ⩾ 0,7 groups. P-values < 0.05 are in bold.
